# A cross-species atlas of the dorsal vagal complex reveals neural mediators of the effects of cagrilintide on energy balance

**DOI:** 10.1038/s42255-026-01539-3

**Published:** 2026-06-08

**Authors:** Mette Q. Ludwig, Bernd Coester, Desiree Gordian, Shad Hassan, Abigail J. Tomlinson, Mouhamadoul Habib Toure, Oliver P. Christensen, Greta Lommi, Anja Moltke-Prehn, Jenny M. Brown, Dylan M. Belmont-Rausch, Sarah Bau, Cagri Bodur, Anika Gowda, Iris Wu, Stace Kernodle, Victoria Dong, Mike Ayensu-Mensah, Paul V. Sabatini, Jae Hoon Shin, Melissa Kirigiti, Kristoffer L. Egerod, Christelle Le Foll, Sofia Lundh, Marina Kjærgaard Gerstenberg, Thomas A. Lutz, Paul Kievit, Anna Secher, Kirsten Raun, Martin G. Myers, Tune H. Pers

**Affiliations:** 1 Novo Nordisk Foundation Center for Basic Metabolic Research, University of Copenhagen, Copenhagen, Denmark; 2 Research and Development, Novo Nordisk, Måløv, Denmark; 3 Departments of Internal Medicine, University of Michigan and Molecular and Integrative Physiology, Ann Arbor, MI USA; 4 Pioneer Centre for SMARTbiomed, University of Copenhagen, Copenhagen, Denmark; 5 Institute of Veterinary Physiology, Vetsuisse Faculty University of Zurich, Zurich, Switzerland; 6 Oregon National Primate Research Center, Oregon Health & Science University, Beaverton, OR USA; 7 One Health Institute, University of Zurich, Zurich, Switzerland; 8 Present Address: Department of Medicine, McGill University, Montreal, Quebec Canada

**Keywords:** Feeding behaviour, Endocrine system and metabolic diseases, Metabolism, Obesity, Neuroscience

## Abstract

Amylin receptor agonists such as cagrilintide represent emerging obesity therapies. To understand mediators of cagrilintide action, we generated a transcriptomics atlas of over 530,000 cells comprising 80 neuronal cell populations across rat, mouse and macaque caudal brainstem, with spatial profiling to map distribution in the rat dorsal vagal complex (DVC). Here we show that cagrilintide regulates two conserved *Calcr*-expressing DVC neuronal populations. While acute cagrilintide treatment alters gene expression in area postrema *Calcr/Ramp3* neurons, chemogenetic activation in rats fails to affect long-term food intake and body weight. In contrast, long-term cagrilintide treatment in rats upregulates prolactin-releasing hormone (*Prlh*) expression in nucleus of the solitary tract *Calcr/Prlh* cells that are conserved across rodents, macaques and humans. Knocking down DVC *Prlh* abrogates the effects of cagrilintide but not semaglutide in rats. Our study provides a cross-species spatially resolved atlas of DVC cell populations and defines *Calcr/Prlh* neurons as mediators of amylin receptor agonist action.

## Abstract (web summary)

To understand which neurons respond to and mediate the action of the amylin receptor agonist cagrilintide, the authors generated a spatially resolved transcriptomics atlas of the caudal brainstem in mice, rats and macaques. Follow-up work in rats identified brainstem *Calcr/Prlh* neurons as mediators of the long-term effects of cagrilintide.

## Main

Amylin analogues reduce food intake and represent promising anti-obesity therapies^1^. Treatment with the long-acting amylin analogue cagrilintide^2^ induces 11.8% weight loss in humans over 68 weeks and co-administering cagrilintide with the glucagon-like peptide-1 (GLP-1) receptor agonist semaglutide produces 22.7% weight loss over 68 weeks (versus 16.1% for semaglutide alone)^3–6^.

Endogenous amylin is co-secreted with insulin from pancreatic beta cells. Whereas amylin acts via the amylin receptor, a complex of CALCR and one of three receptor activity modifying proteins (RAMP1–3), cagrilintide binds the amylin receptor and CALCR with similar potency. To mediate its appetite-suppressing effects, amylin requires an intact brainstem DVC^7–10^. The DVC consists of the area postrema (AP), a circumventricular organ that lies outside the blood–brain barrier; the nucleus of the solitary tract (NTS), the major termination site of vagal afferents; and the dorsal motor nucleus of the vagus (DMV), responsible for parasympathetic outflow to peripheral organs including those of the gut^11^.

DVC *Calcr* neurons contribute to the control of food intake and body weight. Activation of rodent AP and NTS *Calcr* neurons acutely reduces food intake and body weight^12,13^, while AP lesions attenuate acute amylin-induced suppression of food intake^9^. Moreover, knocking down DVC *Calcr* attenuates amylin-mediated activation of DVC neurons and increases meal duration^8^. These roles are consistent with our previous finding that *Calcr*-expressing mouse DVC neurons express transcriptional programmes enriched for genes in obesity-associated genome-wide association study loci^12^. Hence, DVC *Calcr* neurons represent likely candidates for mediating the weight-reducing effects of amylin and its analogues.

Whereas amylin analogues promote profound weight loss in rats, they only mediate a modest weight loss in mice^14^. Although we and others have begun to map the molecular signatures of cell populations in the mouse DVC^12,15,16^, the molecular anatomy of this region remains incompletely defined. Furthermore, cross-species conservation of DVC cell populations is unknown, and the nature of the DVC mediators and mechanisms of action for amylin analogues remain unclear.

To understand DVC conservation and function and the mechanisms of action for amylin analogues, we generated a comprehensive transcriptional atlas of the caudal brainstem comprising >530,400 cells across 90 cell populations from lean and obese mice and rats, and lean rhesus macaques. Spatial profiling of >98,900 cells resolved the anatomical location of 67 neuronal populations, 34 of which reside in the DVC. Most populations were conserved across species, with macaques expressing *Calcr* and *Ramp3* in similar cell populations as rodents. While cagrilintide rapidly modulated gene expression in AP *Calcr/Ramp3* neurons in obese mice and rats, long-term cagrilintide treatment activated NTS *Calcr/Prlh* neurons and upregulated *Prlh* expression specifically in rats, suggesting a role for these cells in the long-term response to cagrilintide. Indeed, activating rat AP *Calcr* neurons suppressed feeding only transiently, whereas NTS *Calcr* neurons decreased food intake and body weight over the long term. *Prlh* expression was required for the long-term suppression of food intake and body weight by cagrilintide in rats.

## Results

### A cross-species transcriptional atlas of the caudal brainstem

**Fig. 1:**
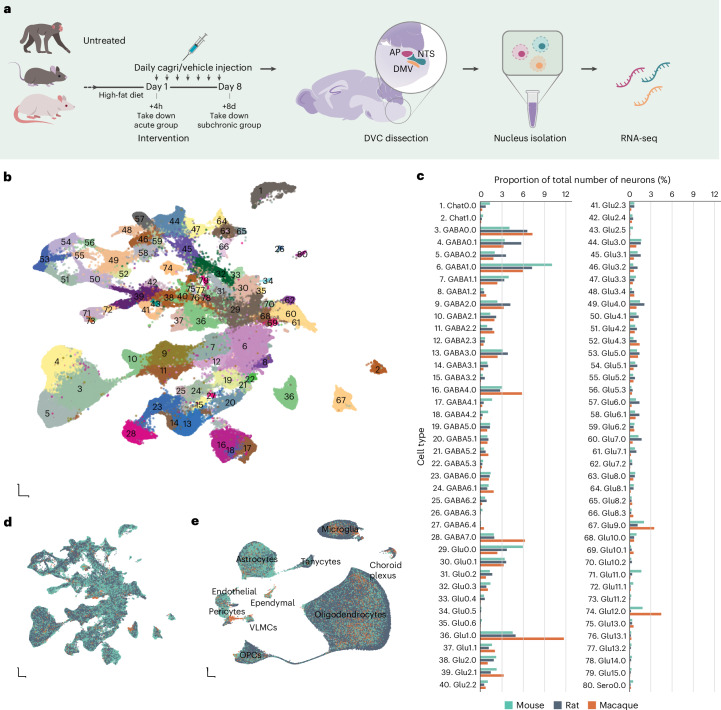
Single-cell transcriptomics cross-species atlas of the DVC. **a**, Overview of the in vivo study with acute and subchronic treatment of DIO rats and mice, plus untreated macaques. **b**, UMAP plot of all 229,129 neurons coloured by cell population. **c**, Proportions of each neuronal cell population contributing to the total number of neurons per species are plotted as corresponding bar graphs. Chat, cholinergic; GABA, GABAergic; Glu, glutamatergic. **d**, UMAP plot of the neurons coloured by species (macaque = 10,111; mouse = 100,354; rat = 118,664). **e**, UMAP of the 301,278 glial cells coloured by species (macaque = 35,618; mouse = 96,062; rat = 169,598). OPCs, oligodendrocyte progenitor cells; VLMCs, vascular and leptomeningeal cells. Source data

To identify species-specific transcriptional response to cagrilintide signalling in the DVC, we treated 30 diet-induced obese (DIO) mice and 42 DIO rats with either a single dose or seven daily doses of cagrilintide or vehicle (subcutaneous or s.c.). To distinguish transcriptional effects from weight loss, subsets of vehicle-dosed mice (*n* = 6) and rats (*n* = 11) were weight-matched to the cagrilintide-treated animals. From these animals, we isolated the caudal brainstem containing the DVC and performed single-nucleus RNA sequencing (snRNA-seq; Fig. 1a, Supplementary Tables 1 and 2 and Supplementary Fig. 1a,b).

**Extended Data Fig. 1:**
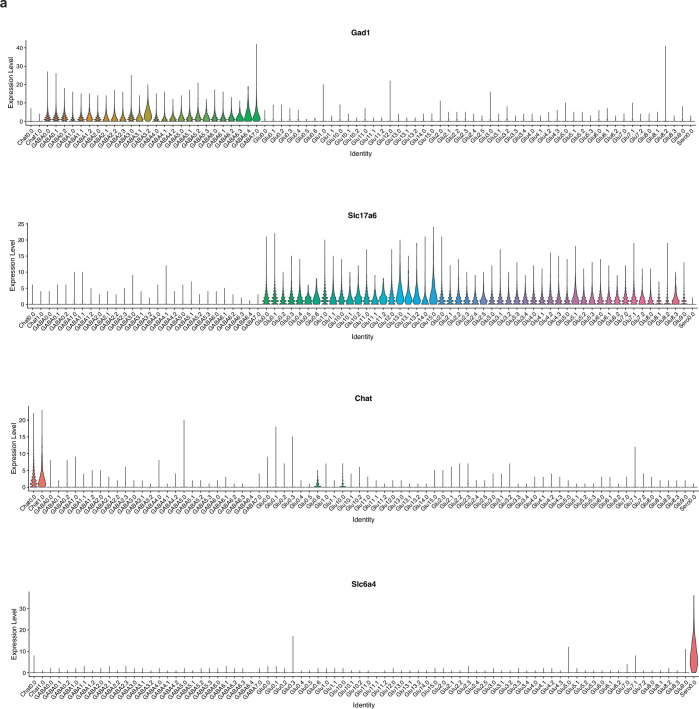
Canonical neurotransmitter marker expression across DVC neuronal populations. **a**, Violin plot showing GABAergic (Gad1), glutamatergic (Slc17a6), cholinergic (Chat) and serotonergic (Slc6a4) marker genes across all neuronal cell populations.

**Extended Data Fig. 2:**
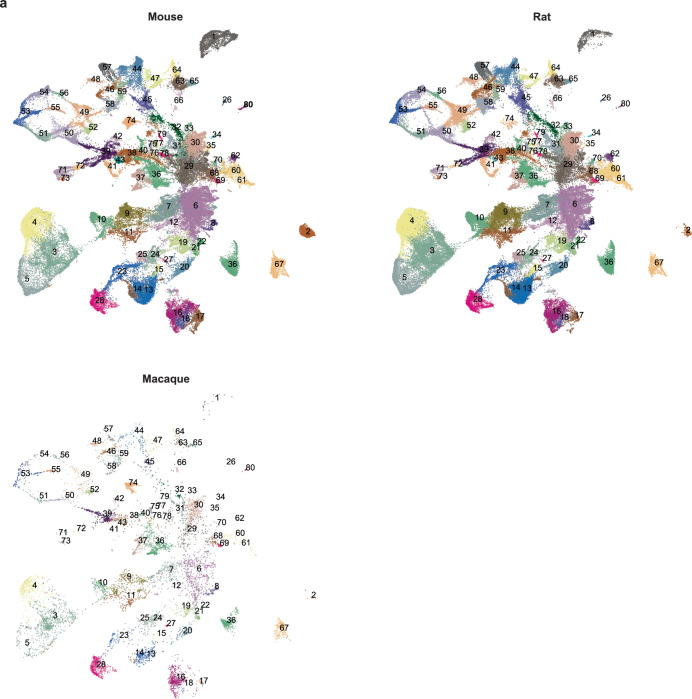
UMAP of neuronal populations split by species. **a**, UMAP of all neuronal clusters colored by cell type and split across each species. Abbreviations: UMAP, uniform manifold approximation and projection.

**Extended Data Fig. 3:**
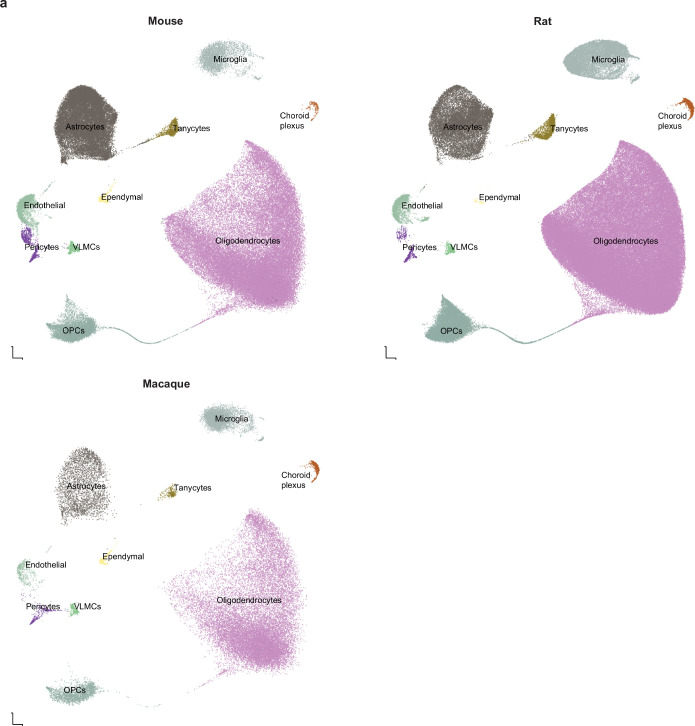
UMAP of glial cell populations split by species. **a**, UMAP of all glia clusters colored by cell type and split across each species. Abbreviations: UMAP, uniform manifold approximation and projection.

To assess the conservation of cell populations and receptor profiles between rodents and primates, we performed snRNA-seq on caudal brainstem tissue from 11 macaques. We split each species atlas into glial cells and neurons, mapped rat and macaque genes to mouse orthologues and integrated across species. To increase coverage of AP cell populations and improve clustering confidence, we also included cells from our previous AP-centric mouse atlas^12^. In total, these efforts yielded an atlas of 301,268 glial and 229,129 neurons (Supplementary Table 3), clustering into 10 glial cell types and 80 neuronal populations with unique marker genes (Fig. 1b–e and Extended Data Figs. 1–3). For consistency, neurons were grouped into three major categories of glutamatergic, GABAergic and cholinergic cells.

**Extended Data Fig. 4:**
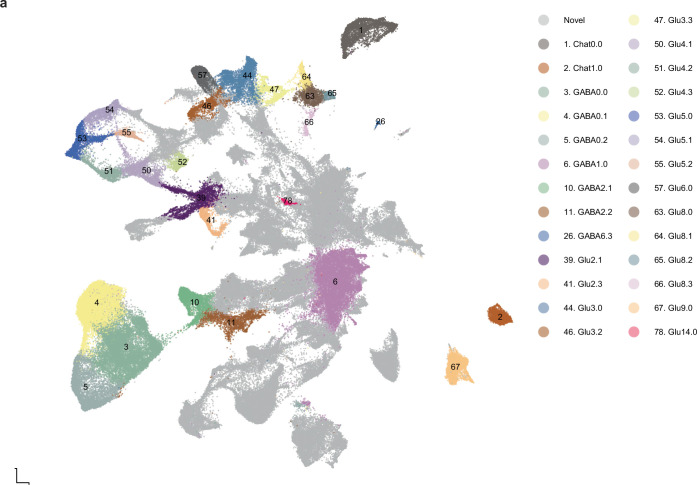
Integration of the previously published mouse DVC atlas into the expanded neuronal atlas. **a**, UMAP of the mouse single-nucleus transcriptomics data from (Ludwig et al. ^12^) included in this atlas plotted over the entire neuronal atlas. Newly discovered regions are colored in grey, clusters from the old atlas are colored in and denoted by their new identity. Abbreviations: UMAP, uniform manifold approximation and projection.

Unlike our previous study^12^, we used a broader dissection strategy encompassing the entire DVC. This identified 53 additional neuronal populations, including 12 previously undescribed DVC-specific cell populations and 41 cell populations likely residing outside the DVC (Extended Data Fig. 4).

Our updated atlas contained 196,416 mouse, 288,262 rat and 45,729 macaque cells. Across neuronal populations, we recovered a median number of >2,750 transcripts (unique molecular identifiers) and >1,650 uniquely detected genes, representing a >1.5-fold increase over our previous AP-centric mouse DVC atlas^12^. In samples not sorted for the neuronal marker NeuN, the proportion of cell types varied across species: neurons constituted 50.2% of mouse cells, 41.2% of rat cells and 22.1% of macaque cells. Conversely, oligodendrocytes made up 61.2% of macaque cells, 42.0% of rat cells and 29.4% of mouse cells, consistent with the reported negative correlation between brain mass and neuron-to-glia ratio across species^17^.

### Spatial mapping of the rat caudal brainstem cell populations

**Fig. 2:**
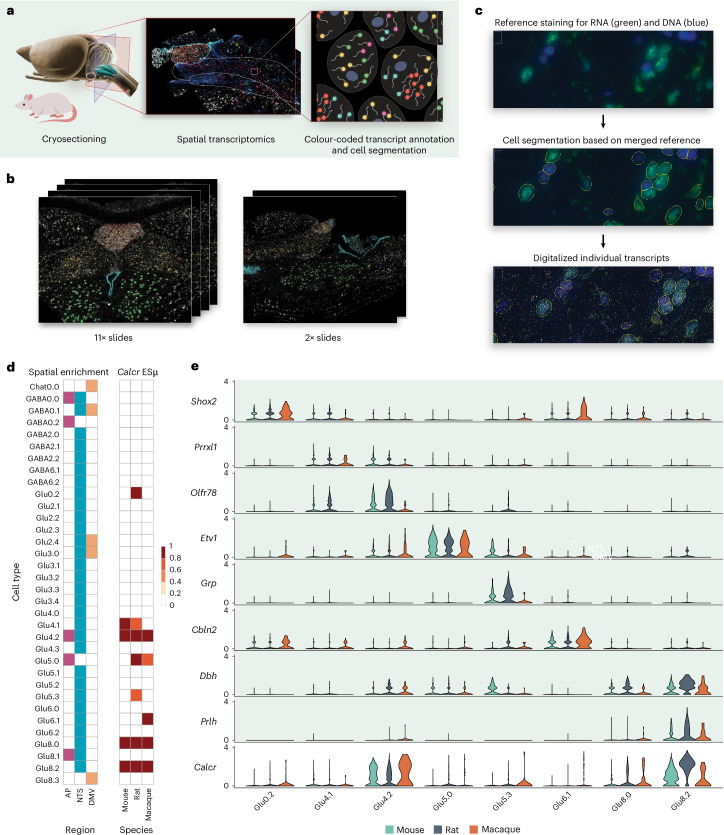
Spatial transcriptomics atlas of the rat DVC. **a**, Overview of experimental set-up for sagittal and coronal sectioning of the rat caudal hindbrain and schematic cell segmentation with transcript annotation. **b**, Overview of raw transcriptomic data with detected transcripts plotted over scanned brain sections. Eleven coronal sections cover a wide range of the DVC and surrounding areas, accompanied by two sagittal sections near the midline. **c**, Detailed view of the cell segmentation process: DAPI staining (blue) and poly(T) staining (green) give a background signal for DNA and RNA in the tissue. The channels are merged and the resulting stack undergoes automated cell segmentation. The transcripts detected through Molecular Cartography are then plotted over it and assigned to each segmented cell. **d**, Left, Spatial enrichment plot showing significant presence of each of the 34 identified DVC cell populations across the AP, NTS and DMV after Fisher’s exact test. Right, *Calcr* expression specificity (CELLEX-based ES_μ_, ranging from zero (low) to one (high)) across the three species. **e**, Violin plots showing (1) expression of genes marking all neuronal populations in which *Calcr* was specifically expressed in at least one species, and (2) *Calcr* expression.

The DVC is a spatially heterogeneous region, and cell populations expressing the same receptors can respond differentially to peripherally administered ligands depending on their location. To assign cell populations to spatial locations within the AP, NTS and DMV, we used Molecular Cartography, a spatial transcriptomics platform based on single-molecule fluorescence in situ hybridization, to profile 13 DVC sections from four lean rats (Fig. 2a and Supplementary Table 4). Due to the longitudinal orientation of the NTS along the anteroposterior axis, we included two sagittal sections near the midline in addition to 11 coronal sections. All sections were manually annotated according to an anatomical atlas^18^ and ordered by their rostrocaudal position (Fig. 2b). Using the combined DAPI and in situ hybridization signal, we estimated cell position and size in each section, and each transcript falling within this segment was assigned to that cell (Fig. 2c). This yielded 98,998 segmented cells across 13 sections, with a median of 196 transcripts and 33 unique genes per cell. Transferring cell population labels from our cross-species snRNA-seq atlas to the spatial transcriptomics atlas revealed distinct distribution patterns for neuronal and glial populations (Supplementary Fig. 2).

**Extended Data Fig. 5:**
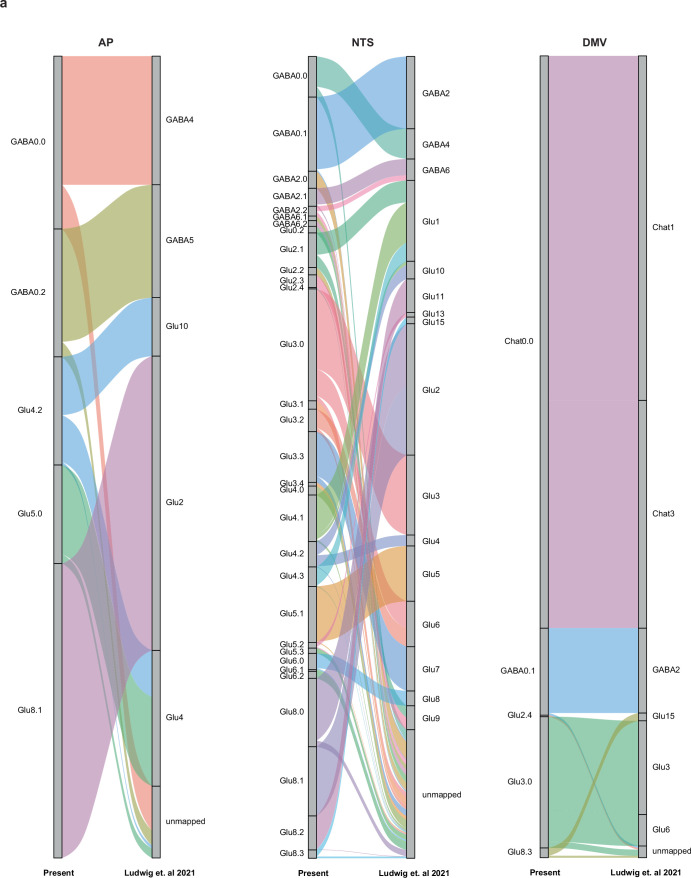
Mapping of DVC neuronal cell populations across anatomical regions to a previously published atlas. **a**, Sankey plot of all neuronal cell types in the DVC mapping to the single cell atlas from Ludwig et al.^12^ across each anatomical region (AP, area postrema; NTS, nucleus of the solitary tract; DMV, Dorsal motor nucleus of the vagus).

We mapped the spatial distribution of 67 of the 80 neuronal populations (83.8%). Of these, 34 (50.7%) mapped within the DVC (Fig. 2d and Supplementary Table 5). All but two populations from our previous AP-centric DVC atlas mapped to the present cross-species atlas (Extended Data Fig. 5). The combined cross-species snRNA-seq and spatial atlases are accessible for download and via an interactive website (see ‘Data availability’).

Building on this spatial annotation of cell populations, we focused on *Calcr*-expressing neurons. We assessed *Calcr* expression across all DVC populations and used CELLEX^19^ to compute expression specificity (ES_μ_; ranging from 0 to 1) for each species. This analysis revealed that 8 of the 34 neuronal populations expressed *Calcr* (ES_μ_ > 0.8) in at least one species. While some populations expressed *Calcr* in only one species (for example, Glu6.1 in macaques), three neuronal populations expressed *Calcr* in all species (Fig. 2d and Supplementary Table 6).

### Characterization of conserved *Calcr*-expressing cell populations

**Fig. 3:**
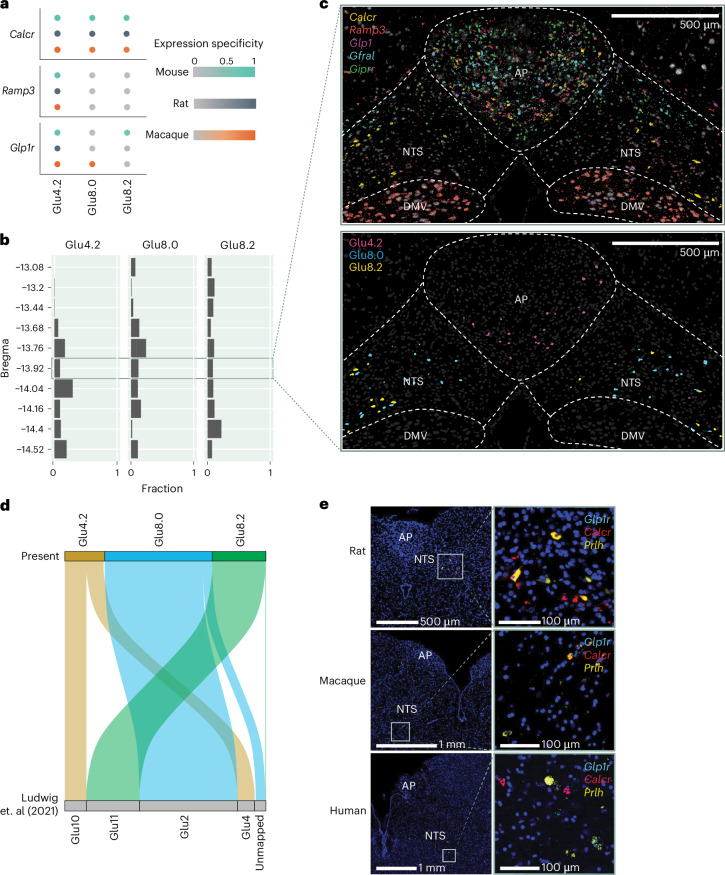
Conservation of cell populations across mice, rats and macaques, and spatial location of *Calcr*-positive cell populations in the rat. **a**, Expression of *Calcr* and *Ramp3* (the components of the heterodimeric Amy3R) and *Glp1r* across conserved *Calcr*-expressing neuronal cell populations. Only cell populations expressing *Calcr* with high confidence (ES_µ_ > 0.8) in all species and successfully mapped to the rat spatial atlas are included. **b**, Frequency of each given cell population through varying bregma levels based on data derived from the 11 coronal sections in the rat spatial atlas. **c**, Upper panel shows a DAPI+PolyT scan with detected *Calcr*, *Ramp3*, *Glp1r*, *Gfral* and *Gipr* transcripts plotted to represent the raw spatial transcriptomics data (Resolve Polylux plugin to ImageJ). Lower panel shows a segmented, digitalized and annotated spatial dataset representing the previously identified three cell population annotations transferred from the single-cell atlas. Plot from the RShiny Ratlas (coronal section bregma −13.92). **d**, Alluvial diagram mapping *Calcr*-positive cells to our previous DVC atlas. **e**, Representative images for RNAScope multiplexed in situ hybridization data for *Prlh*, *Calcr* and *Glp1r* in rat (top), macaque (middle) and human (bottom) brain tissue. Right panels show digital zooms of boxed regions in left panels. *Calcr*, calcitonin receptor; *Ramp3*, Receptor activity modifying protein 3; *Glp1r*, glucagon-like peptide-1 receptor, *Gfral*, GDNF family receptor alpha like; *Gipr*, gastric inhibitory polypeptide receptor. Source data

**Extended Data Fig. 6:**
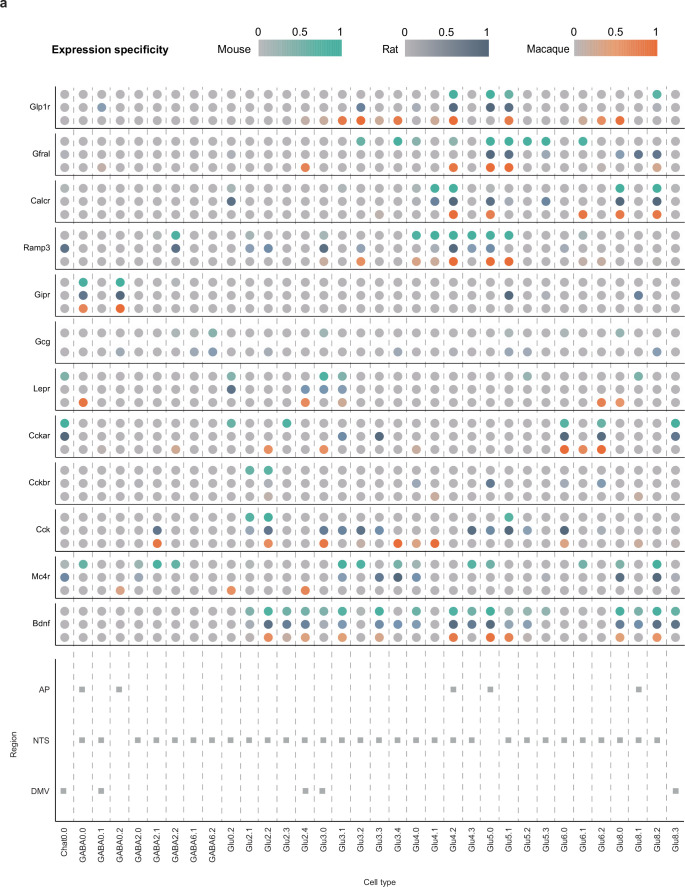
Cross-species expression of key neuropeptide and receptor genes across DVC cell populations. **a**, Expression of *Bdnf*, *Mc4r*, *Cck*, *Cckbr*, *Cckar*, *Lepr*, *Gipr*, *Ramp3*, *Calcr*, *Gfral* and *Glp1r* across DVC cell populations in mouse, rat and macaque, represented by predicted expression specificity ESmu^28^. *Gcg* was not expressed in macaque and has been omitted. Bottom panel shows the anatomical regions of each cell population.

**Extended Data Fig. 7:**
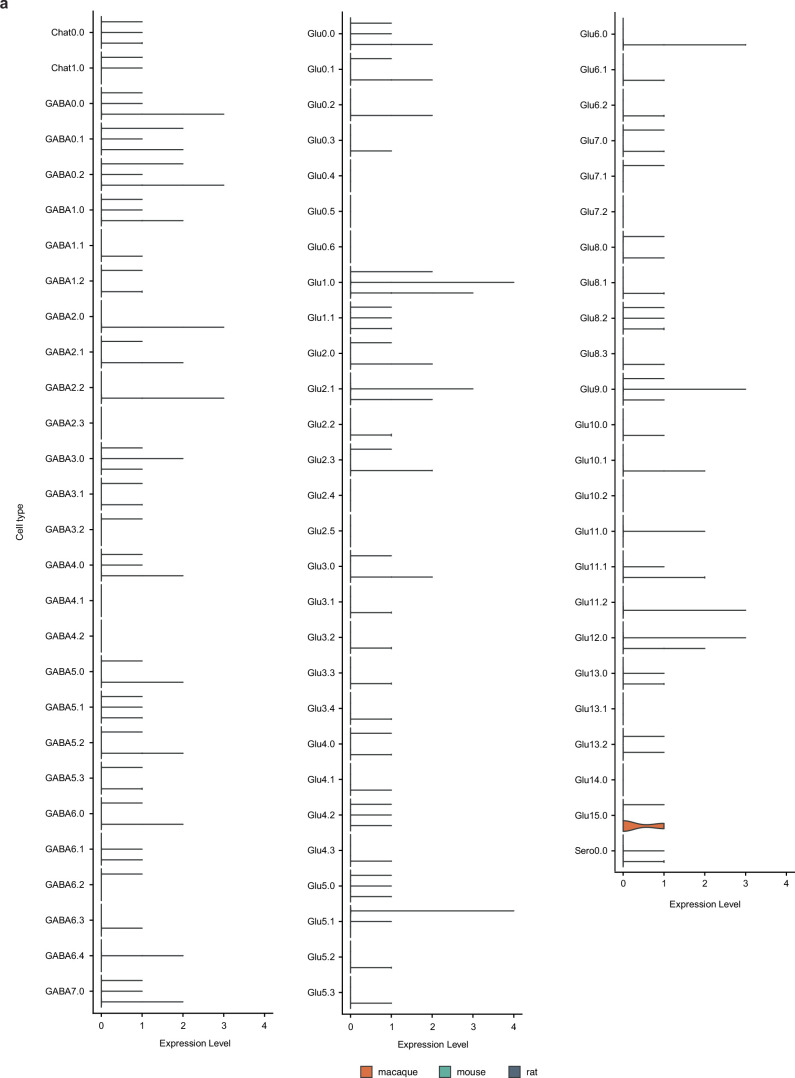
Expression of *Ramp1* across DVC neuronal cell populations. **a**, Violin plot showing expression of *Ramp1* across all DVC neuronal cell populations in mouse, rat, and macaque.

**Extended Data Fig. 8:**
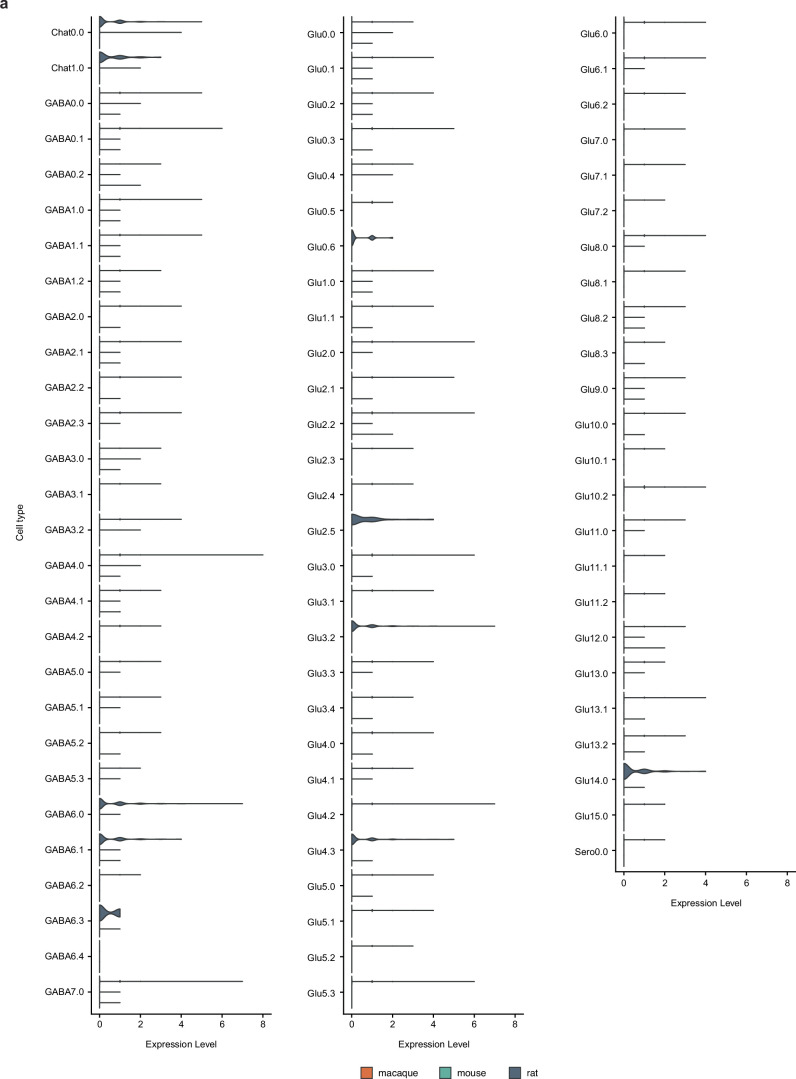
Cross-species expression of *Ramp2* across DVC neuronal cell populations. **a**, Violin plot showing expression of *Ramp2* across all DVC neuronal cell populations in mouse, rat, and macaque.

**Extended Data Fig. 9:**
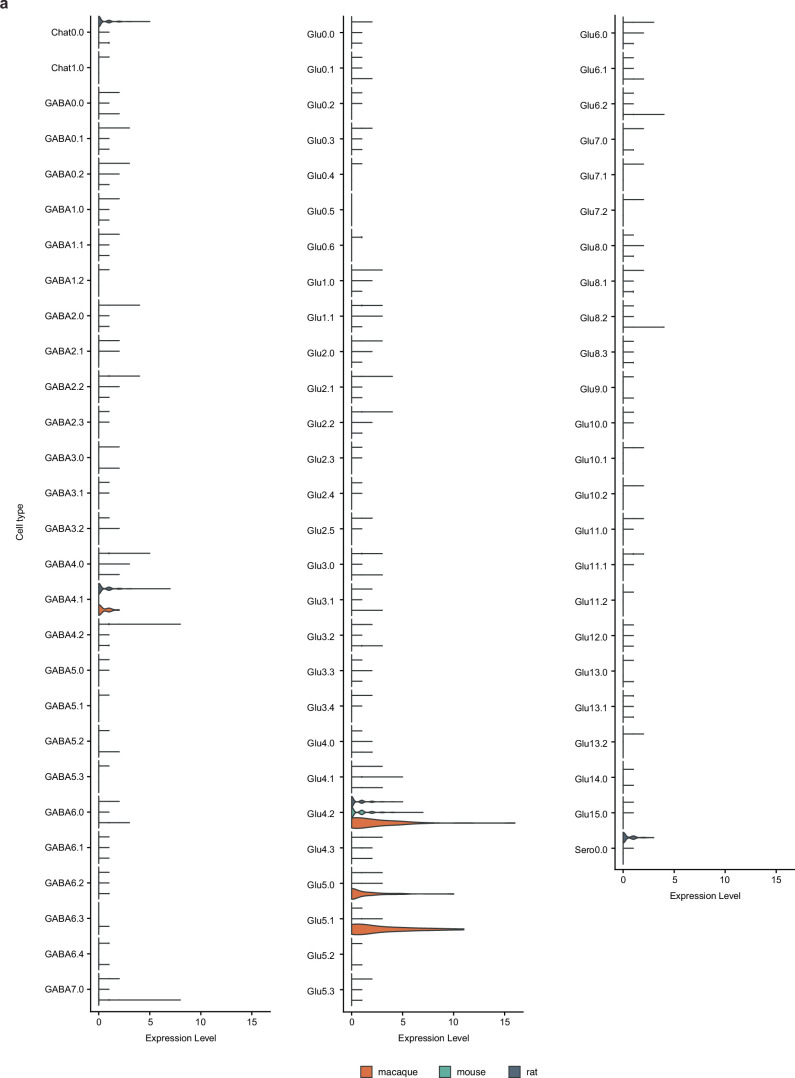
Cross-species expression of *Ramp3* across DVC neuronal cell populations. **a**, Violin plot showing expression of *Ramp3* across all DVC neuronal cell populations in mouse, rat, and macaque.

**Extended Data Fig. 10:**
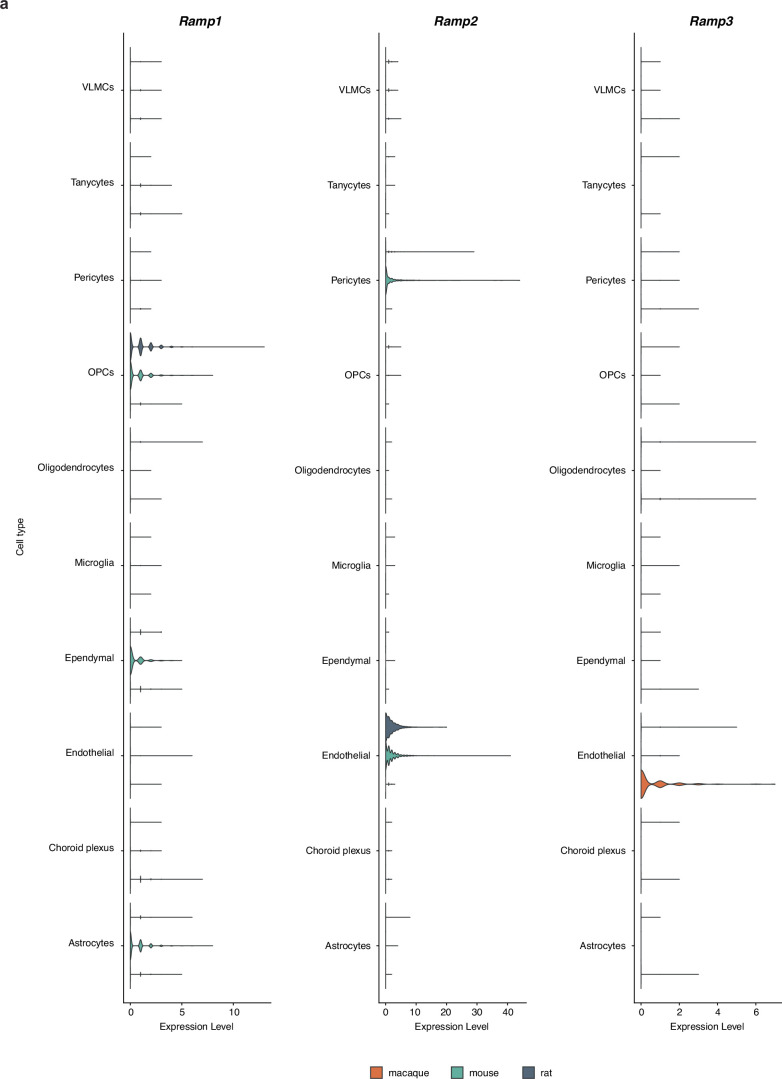
Cross-species expression of *Ramp1*, *Ramp2* and *Ramp3* across DVC glial cell populations. **a**, Violin plot showing expression of *Ramp1/2/3* across all DVC glia cell populations in mouse, rat, and macaque.

The Glu4.2, Glut8.0 and Glu8.2 populations expressed *Calcr* across all three species, although Glu4.2 and Glu8.2 contained more *Calcr*-expressing cells than Glu8.0 (Fig. 3a). Of these, Glu4.2 also co-expressed *Ramp3* across all species (see Extended Data Figs. 6–10 for *Ramp3* and other peptide receptor expression across populations). We mapped the distribution of these three populations in the rat spatial data, and our previous mouse DVC atlas^12^ (Fig. 3b–d and Extended Data Fig. 5). The Glu4.2 *Calcr/Ramp3* neurons resided predominantly in the AP, although some cells mapped to the medial NTS. They correspond to the previously reported AP GLU10 *Calcr/Ramp3* population combined with the *Glp1r/Casr/Tnfrsf11b*-expressing subpopulation (GLU4a in ref. ^20^) of the larger mouse AP GLU4 *Glp1r/Gfral* neuronal population. Indeed, in rats, *Glp1r*-expressing neurons and Glu4.2 *Calcr/Ramp3* neurons were transcriptionally distinct (Supplementary Fig. 3). Furthermore, in *Calcr*^*Cre*^ rats injected with adeno-associated virus (AAV)-DIO-hM3Dq-mCherry in the AP, essentially all mCherry-identified AP *Calcr*^*Cre*^ neurons contained CALCR but not GLP1R immunoreactivity (we lack a reliable reporter allele for *Calcr*^Cre^ in rats; Supplementary Fig. 4), suggesting that rat AP CALCR and GLP1R neurons are distinct. These findings suggest that Glu4.2 may contain two different (albeit transcriptionally related) neuronal subtypes. However, *Glp1r* and *Calcr* expression did not segregate in mouse Glu4.2 *Calcr/Ramp3* neurons. Although transient developmental receptor expression remains a possibility, staining in *Calcr*^*Cre*^ and *Glp1r*^*Cre*^ mice demonstrated that roughly half of the AP *Calcr* cells contain GLP1R immunoreactivity, and vice versa (Supplementary Fig. 5). Hence, while *Glp1r* and *Calcr* expression mark distinct subsets of rat AP Glu4.2 *Calcr/Ramp3* neurons, these receptors are less segregated in mouse AP Glu4.2 *Calcr/Ramp3* cells.

Glu8.0 and Glu8.2 mapped to distinct previously annotated mouse NTS neuronal populations, GLU2 and GLU11, respectively. In rats, Glu8.0 neurons localized to the medial and intermediate NTS near the AP (Fig. 3c). Glu8.2 spanned medial and lateral NTS and along its entire rostrocaudal extent.

In mice, we previously found that the NTS Glu8.2 *Calcr/Prlh* population includes *Glp1r*-expressing neurons. In our current snRNA-seq dataset, the Glu8.2 *Calcr/Prlh* population contains cells that express *Glp1r*, albeit at low levels in rats and macaques (Supplementary Figs. 6–8). Due to the short length of the *Prlh* transcript, we could not include this probe in our spatial transcriptomics panel. Thus, we used RNAscope in situ hybridization to determine the location and receptor expression pattern across rats, macaques and humans (Fig. 3e). This analysis confirmed the presence of *Prlh/Calcr/Glp1r* co-expressing neurons in the NTS of all three species, as well as *Prlh*-expressing cells with neither receptor present. *Prlh* and *Calcr* were extensively co-expressed in rats, while macaques and humans exhibited more *Prlh/Glp1r* co-expression (Supplementary Fig. 9).

### Engagement of NTS Glu8.2 *Calcr/Prlh* neurons by cagrilintide in rats but not in mice

**Fig. 4:**
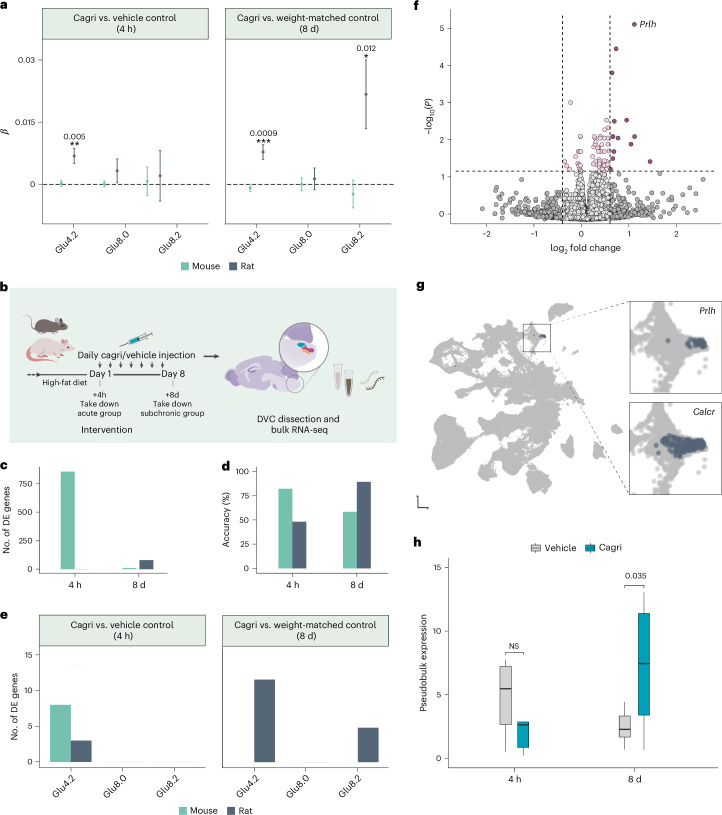
Cagrilintide activates NTS *Prlh*-expressing neurons and promotes their expression of *Prlh* in rats but not in mice. **a**, Change in activity of *Fos*-regulated genes in conserved *Calcr* neurons in mice and rats between exposure to cagrilintide and vehicle control followed by snRNA-seq of the DVC. Values are the mean estimate ± s.e. *P < 0.05 versus vehicle; linear mixed-effects model with sample included as covariates (mice *n* = 6; rat cagrilintide subchronic and vehicle acute *n* = 10; rat cagrilintide acute and weight-matched subchronic *n* = 11), followed by a Benjamini–Hochberg (BH)-adjusted two-tailed least-squares means *t*-test. **b**, Overview of the rat and mouse in vivo studies used for bulk RNA-seq. **c**, Number of differentially expressed (DE) genes between cagrilintide-administered and control animals in the acute (4 h) and subchronic (7 day) groups in the bulk RNA-seq experiment. **d**, Accuracy of regularized logistic regression classifiers to discriminate between cagrilintide-administered and control animals. **e**, Cell population-specific differential gene expression in mice and rats under the same experimental paradigm of cagrilintide treatment versus vehicle. **f**, Volcano plot of differentially expressed genes for subchronically cagrilintide-administered and weight-matched control rats. Statistical significance for the treatment effect was assessed using two-tailed Wald tests. *P* values were adjusted for multiple comparisons using the BH false discovery rate (FDR) method. Red colour indicates a log_2_ fold change > 0.5 and BH-adjusted DESeq2 *P* < 0.05. Five data points with a log_2_ fold change > 3 were omitted from this graph to avoid compressing most results along the *x* axis. **g**, Feature plot of normalized *Prlh* expression in the extended DVC atlas (Fig. 1b; cluster 65) marking a specific cell population. *Calcr* feature plot of the magnified UMAP region is shown as well. **h**, Expression of *Prlh* in Glu8.2 (*Calcr*/*Prlh*) neurons from rats in the acute (vehicle *n* = 8; cagrilintide *n* = 5) and subchronic cagrilintide treatment (*n* = 5) groups. Acute correlations: vehicle (min., 0.5; max., 20.8; median, 5.4) and cagrilintide (min., 0.2; max., 8.6; median, 2.6). Subchronic correlations: vehicle (min., 0.7; max., 4.4; median, 2.2) and cagrilintide (min., 0.6; max., 13.0; median, 7.4). *P < 0.05 versus vehicle; two-tailed Wald test. DE, differential expression; NS, not significant. Source data

To identify cellular mediators of cagrilintide action, we used expression of the immediate early gene *Fos* and its downstream target genes (the *Fos* regulon) as a transcriptional proxy for neuronal activation. We focused on *Calcr*-expressing neuronal populations as potential first-order cagrilintide targets. In rats, both acute (4-h) and subchronic (7-day) cagrilintide treatment increased predicted *Fos* regulon activity in Glu4.2 *Calcr/Ramp3* cells (adjusted *P* value (*P*_adj_) = 0.037 and *P*_adj_ = 0.026, respectively; Fig. 4a). Subchronic cagrilintide treatment also increased *Fos* regulon expression in the Glu8.2 NTS *Calcr/Prlh* population in rats (*P*_adj_ = 0.026; Fig. 4a). We detected no increase in *Fos* regulon expression in Glu8.0 neurons under any conditions. Although FOS is only one proxy for neuronal activity-dependent gene expression, these results suggest that acute cagrilintide treatment in rats activates Glu4.2 *Calcr/Ramp3* neurons, whereas subchronic treatment additionally engages Glu8.2 NTS *Calcr/Prlh* neurons, with no evidence for Glu8.0 activation under any condition. We detected no *Fos* regulon activation in mouse populations; this may reflect the more rapid onset (1 h) and decline of cagrilintide-induced FOS accumulation in mice relative to rats^21^.

To improve detection of differentially expressed genes, we treated additional DIO rats and mice with cagrilintide and performed bulk RNA-seq on DVC tissue (Fig. 4b). While this method cannot provide single-cell resolution, it detects changes in gene expression with greater sensitivity than snRNA-seq. Acute (4-h) treatment with cagrilintide induced 856 differentially expressed genes in mice (compared to vehicle; *P*_adj_ < 0.05). We detected no differentially expressed genes in acutely treated rats (although 423 genes were nominally significant). This finding may reflect the slower onset of cagrilintide-mediated DVC changes in rats compared to mice.

Conversely, subchronic (7-day) cagrilintide treatment altered only 10 genes in mice, whereas 79 genes where differentially expressed in rats (Fig. 4c and Supplementary Table 9). Gene Ontology (GO) enrichment analysis of these 79 genes identified processes related to synapse pruning and microglial activation, including interleukin-6 production and chemotaxis (Supplementary Table 10). Consistent with these findings, FOS immunostaining in cagrilintide-treated mice showed robust signal 2 h and 6 h after injection but no FOS expression following subchronic treatment (Supplementary Fig. 10). In contrast, rat DVC displayed increased FOS following both acute and subchronic cagrilintide treatment (Supplementary Fig. 11).

We next quantified the robustness of the differential gene expression in rats at the subchronic (7-day) time point. We trained regularized logistic regression models using leave-one-out cross-validation to predict treatment group from gene expression. In the acute study, classifiers discriminated cagrilintide-treated from control mice with 82.1% accuracy (23/28 correct; empirical *P*_adj_ = 0.016) but performed poorly in rats (48.1% accuracy). For the subchronic study, classifiers failed in mice (58.3% accuracy) but achieved 89.3% accuracy in rats (25/28 correct; empirical *P*_adj_ = 0.016; Fig. 4d). These results indicate that, despite relatively few differentially expressed genes at the subchronic time point, cagrilintide-treated rats can be reliably distinguished from controls based on their transcriptional profile.

To identify genes induced by subchronic cagrilintide treatment in the snRNA-seq data, we performed differentially expression analysis across the three conserved *Calcr*-expressing neuronal populations. The pattern confirmed our findings: cagrilintide acutely altered gene expression in Glu4.2 *Calcr/Ramp3* neurons in both mice and rats, while subchronic treatment affected both Glu4.2 *Calcr/Ramp3* and Glu8.2 NTS *Calcr/Prlh* neurons only in rats (Fig. 4e and Supplementary Table 11). Consistent with the snRNA-seq analysis, bulk RNA-seq identified no differential gene expression mapping to Glu8.0 neurons following cagrilintide treatment.

In subchronic bulk RNA-seq, *Prlh* was the most differentially expressed gene in rats (log_2_ fold change = 1.01, *P*_adj_ = 5.6 × 10^−6^; Fig. 4f); however, *Prlh* was unchanged in mice. Glu8.2 NTS *Calcr/Prlh* neurons represent the only DVC cell population to express *Prlh* (Fig. 4g; cluster 65 in Fig. 1b). Examining *Prlh* in rat Glu8.2 NTS *Calcr/Prlh* neurons in the snRNA-seq data confirmed its upregulation following subchronic cagrilintide treatment (log_2_ fold change = 1.60, *P* = 0.038; Fig. 4h).

Together, these two different transcriptomics approaches applied to two independent in vivo studies suggest the cagrilintide acutely activates Glu4.2 *Calcr/Ramp3* neurons in mice and rats. Subchronic treatment additionally activates Glu8.2 NTS *Calcr/Prlh* neurons and upregulates *Prlh* expression specifically in rats, but not in mice. We found no evidence for the regulation of Glu8.0 neurons by cagrilintide under any treatment conditions.

### Cagrilintide effects are likely neuronal and not obesity dependent

To investigate whether cagrilintide reverses obesity-associated changes or through distinct mechanisms, we identified differentially expressed genes between the chow-fed control group and DIO animals (acute and subchronic vehicle treatment; Supplementary Table 12). Notably, none of the conserved *Calcr*-expressing populations were significantly altered by DIO. We also detected no alteration in *Prlh* expression in Glu8.2 NTS *Calcr/Prlh* neurons between chow-fed and DIO rats or mice. These findings suggest that cagrilintide treatment does not simply reverse obesity-induced transcriptional changes but engages alternative, obesity-independent mechanisms.

Given the microglia-related GO terms enriched among subchronic differentially expressed genes in rats, we examined whether glial cells contributed to the effects of cagrilintide. Mapping subchronic rat bulk differentially expressed genes to cell populations revealed some overlap with microglia (Supplementary Fig. 12). However, we detected no *Calcr* expression in rodent microglia or other glial cell populations in either the snRNA-seq or spatial transcriptomics data. The *Fos* regulon was also not upregulated in microglia following cagrilintide treatment (Supplementary Fig. 13). While these observations do not fully exclude a role for microglia in the subchronic response, they suggest that neurons represent the primary mediators of cagrilintide action.

### AP *Calcr* neuron activation acutely decreases food intake but not long-term body weight

**Fig. 5:**
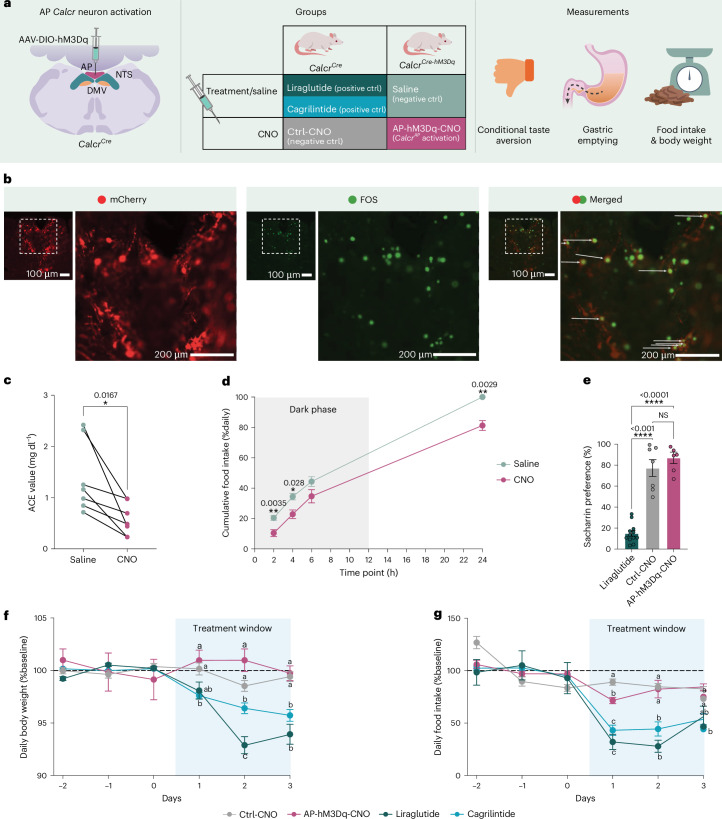
DREADD-mediated activation of rat AP *Calcr*^*Cre*^ neurons acutely decreases food intake but fails to mediate the long-term suppression of body weight or food intake. **a**, Schematic showing stereotaxic injections of activating DREADD virus into the AP. **b**, Representative image of the DVC of a CNO-treated Calcr^AP-Dq^ rat showing DsRed (mCherry)-IR (red, left), FOS-IR (green, middle) and merged images (right). Arrowheads indicate examples of colocalized cells. Scale bar, 200 μm. Right panels show digital zooms of the boxed areas in the left panels. **c**, Calcr^AP-Dq^ rats were treated with saline or CNO 30 min before gavage with acetaminophen (ACE) in saline. Blood was collected 15 min later and assayed for ACE (*n* = 7 per group). **d**, Calcr^AP-Dq^ rats were treated with saline or CNO (1 mg per kg body weight, i.p.) 30 min before the onset of the dark cycle and food intake was measured at the indicated times thereafter. Food intake for each animal was normalized to their 24-h intake following saline administration (*n* = 8 per group). **e**, Rats were exposed to saccharine in drinking water 2 h before the administration of liraglutide or CNO (control rats) or CNO (Calcr^AP-Dq^ rats). Two days later the animals were given a choice between water and saccharine in water and the consumption of each was measured for 4 h. Ratio of saccharine to water consumed is plotted for each animal. All values are plotted as the mean ± s.e.m. (*n*_liraglutide _= 12, *n*_CNO_ = 7, *n*_hM3Dq-CNO_ = 6). **f**,**g**, Food intake and body weight were measured each day during 3 days of saline injection (i.p., twice daily), followed by treatment with (shaded area): CNO (1 mg per kg body weight, i.p., twice daily) for control and Calcr^AP-Dq^ rats and saline (morning) and liraglutide (evening, 100 μg per kg body weight, i.p.) and cagrilintide (evening, 10 nmol per kg body weight) for 3 days. Body weight and food intake are shown normalized to average values for each animal during the initial 3 days of saline treatment (*n*_liraglutide _= 14, *n*_CNO_ = 8, *n*_hM3Dq-CNO_ = 8, *n*_cagrilintide_ = 19; two-way analysis fo variance (ANOVA) with Sidak’s multiple-comparisons test with values in the mean ± s.e.m.). **c**, *P = 0.0167 by a two-tailed *t*-test, no error bars. **d**, Two-way ANOVA with values in the mean ± s.e.m., followed by Sidak’s multiple-comparisons test. *P < 0.05; **P < 0.01; ***P < 0.001; ****P < 0.0001. **e**, One-way ANOVA, *P* < 0.0001 versus liraglutide. **f**,**g**, ‘a, b, c’ notations indicate significantly different data points at each *x*-axis value, *P* < 0.05 after Tukey’s multiple-comparisons test. ACE, acetaminophen; DREADD, designer receptors activated only by designer drugs; hM3Dq, human M3 muscarinic DREADD, Gq-coupled signalling. Source data

To determine whether Glu4.2 *Calcr/Ramp3* neurons mediate long-term suppression of body weight in rats, we injected a Cre-dependent AAV expressing hM3Dq-mCherry into the AP of *Calcr*^*Cre*^ knock-in rats, enabling chemogenetic activation of AP *Calcr* neurons with clozapine-*N*-oxide (CNO; Fig. 5a). We confirmed AP-restricted hM3Dq-mCherry expression by examining mCherry immunoreactivity (Fig. 5b), excluding animals with misplaced injections or viral spread into neighbouring regions. Acute CNO treatment (90 min) induced FOS accumulation in mCherry-labelled AP neurons of Calcr^AP-Dq^ rats, confirming CNO-dependent activation (Fig. 5b).

Activating AP *Calcr* neurons acutely decreased gastric emptying (Fig. 5c), and a single CNO injection immediately before the onset of the dark cycle reduced food intake in Calcr^AP-Dq^ rats by over 50% over the first 2 h of monitoring (consistent with our previous findings^13^; Fig. 5d). This suppression continued over subsequent measurements but waned at later times, such that cumulative food intake was only approximately 15% lower than saline-treated controls by 24 h. Hence, suppression of food intake by acute activation of AP *Calcr* neurons is strongest shortly after activation, similar to CALCR ligand treatment at dark-phase onset^8^.

Despite the strong acute suppression of food intake, CNO treatment failed to promote conditioned taste avoidance (a measure of the perception of gastrointestinal malaise) in Calcr^AP-Dq^ rats (Fig. 5e), consistent with previous findings in mice^16^. This suggests that AP *Calcr* neurons mediate non-aversive suppression of gastric emptying and food intake.

To determine whether AP *Calcr* neurons suppress food intake and body weight long term, we treated Calcr^AP-Dq^ rats with CNO twice daily (30–60 min before dark and light cycle onset) for 3 days and compared their food intake to that of control animals treated with CNO, cagrilintide or the GLP1R agonist liraglutide (Fig. 5f,g and Supplementary Fig. 14). Cagrilintide and liraglutide each significantly suppressed food intake and body weight over the 3 days in control rats. However, CNO promoted only a small decrease in food intake in Calcr^AP-Dq^ rats during the first 24 h of treatment (as in Fig. 5d) and failed to reduce food intake over subsequent days or body weight at any time point. To determine whether this lack of long-term effect might reflect desensitization of CNO-dependent AP *Calcr* neuron activation, we treated a separate set of Calcr^AP-Dq^ rats with CNO (1 mg per kg body weight, intraperitoneally (i.p.) twice daily) for 72 h before perfusion and examined mCherry and FOS immunoreactivity. This demonstrated continued FOS in mCherry-labelled AP *Calcr* neurons following prolonged CNO treatment (Supplementary Fig. 14), indicating that the lack of long-term effect does not result from the tachyphylaxis of AP *Calcr* neuron activation.

Thus, activating AP *Calcr* neurons in rats rapidly decreased food intake and gastric emptying, but was insufficient to mediate the long-term suppression of food intake and body weight. Hence, long-term responses to cagrilintide in rats most likely also require engagement of neural systems beyond the Glu4.2 *Calcr/Ramp3* population.

Although we achieved complete AP *Calcr* neuron ablation (via Cre-dependent activated CASPASE3) in only a small number of rats, these animals retained the expected decrease in food intake following cagrilintide treatment (Supplementary Fig. 15). Together with our transcriptomic analysis (Figs. 3 and 4), this finding suggests that Glu4.2 *Calcr/Ramp3* neurons contribute minimally to the subchronic cagrilintide responses. Instead, Glu8.2 NTS *Calcr/Prlh* neurons may mediate the long-term suppression of food intake and body weight by cagrilintide.

### A key role of NTS *Prlh* in the suppression of food intake and body weight by cagrilintide

To test whether NTS *Calcr/Prlh* neurons mediate long-term suppression of food intake and body weight, we injected Cre-dependent expression of hM3Dq-mCherry into the NTS of *Calcr*^*Cre*^ rats, permitting the CNO-dependent activation of NTS *Calcr* neurons (Supplementary Fig. 16). As expected based upon the response in mice^10^, CNO reduced food intake and body weight over several days of treatment.

To test whether Glu8.2 NTS *Calcr/Prlh* neurons are required for cagrilintide-induced weight loss, we initially transduced the NTS of *Calcr*^*Cre*^ rats with AAV-DIO-TetTox-GFP to silence release of neurotransmitters from NTS *Calcr* neurons. While these rats recovered normally over the week following surgery, all animals receiving intra-NTS AAV-DIO-TetTox-GFP died over the subsequent 2–3 weeks. Since silencing of NTS *Calcr* neurons in mice causes no lethality^13^, these findings suggest that a rat-specific *Calcr-*expressing NTS population (for example, Glu0.2; Fig. 2d) is required for survival.

**Fig. 6:**
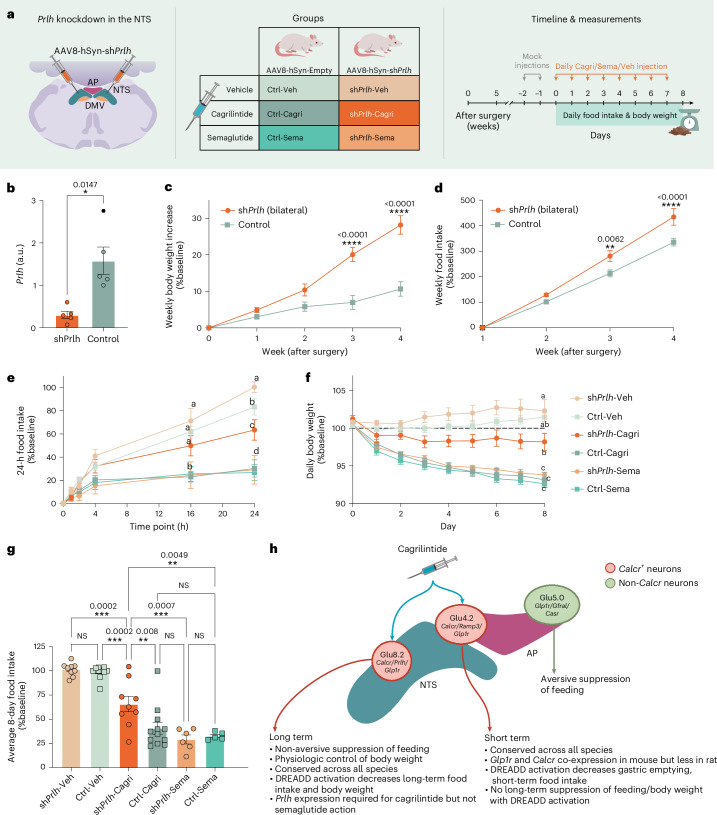
*Prlh* knockdown in the rat NTS attenuates cagrilintide-induced body weight loss. **a**, Schematic of AAV-sh*Prlh* knockdown strategy. **b**, DVC *Prlh* normalized to control gene expression in sh*Prlh* and control rats (sh*Prlh* *n* = 5; control *n* = 5), expressed in arbitrary units or a.u., two-sided *t*-test. **c**,**d**, Body weight and cumulative food intake (normalized to presurgical baseline) during the first 5 weeks after surgery (sh*Prlh* *n* = 9; control *n* = 12). **e**, sh*Prlh* or control rats (sh*Prlh* *n* = 9; control *n* = 12) were treated with vehicle, cagrilintide (10 nmol per kg body weight, i.p.) or semaglutide (10 nmol per kg body weight, i.p.) 30 min before the onset of the dark cycle, and food intake was measured at the indicated times thereafter. **f**,**g**, Body weight and averaged cumulative food intake during subchronic treatment with vehicle, cagrilintide (10 nmol per kg body weight, i.p., once daily) or semaglutide (10 nmol per kg body weight, i.p., once daily) in control rats and sh*Prlh* rats. **h**, Schematic summary of findings and model showing mediators of cagrilintide action. **e**–**g**, Data are shown normalized to average values for each animal over the 3 days before treatment (sh*Prlh*_vehicle_ *n* = 9; sh*Prlh*_cagrilintide_ *n* = 9; sh*Prlh*_semaglutide_ *n* = 5; ctrl_vehicle_ *n* = 12; ctrl_cagri_ *n* = 12; ctrl_sema_ *n* = 5). All values are plotted as the mean ± s.e.m. **b**, *P = 0.0147 by a two-tailed *t*-test with Welch’s correction. **c**,**d**, *P < 0.05; **P < 0.01; ***P < 0.001; ****P < 0.0001 after two-way ANOVA with Sidak’s multiple-comparisons test. **e**–**g**, ‘a, b, c’ notations indicate significantly different data points at each *x*-axis value, *P* < 0.05 after two-way ANOVA with Tukey’s multiple-comparisons test. Source data

Subchronic cagrilintide treatment in rats increased the expression of *Prlh*, which is unique to Glu8.2 NTS *Calcr/Prlh* neurons and associated with energy balance in mice^13,22^. Therefore, we used an AAV expressing a *Prlh*-targeting short hairpin RNA (sh*Prlh*) to knock down *Prlh* expression in the NTS. We injected AAV-sh*Prlh* or AAV-shScrambled (control) into the NTS of DIO rats (Fig. 6a), targeting coordinates corresponding to Glu8.2 NTS *Calcr/Prlh* neurons (Fig. 3c,d). After completion of these studies, we dissected the DVC and quantified *Prlh* expression by quantitative PCR with reverse transcription (RT–qPCR), normalizing *Prlh* expression to *Grpr* (another Glu8.2-specific gene) to control for dissection variability (Fig. 6b).

NTS sh*Prlh* rats showed increased food intake and body weight during the first 5 weeks after surgery (Fig. 6c,d), consistent with the known role of NTS *Prlh* in the restraint of food intake and body weight gain in DIO mice^13,22^. We then acutely treated these rats with cagrilintide or semaglutide and monitored food intake for 24 h. Cagrilintide treatment failed to suppress food intake over 24 h in NTS sh*Prlh* rats to the same extent as in control animals, whereas semaglutide responses were unaltered (Fig. 6e). We also treated NTS sh*Prlh* and control rats subchronically with cagrilintide or semaglutide and measured their food intake and body weight daily (Fig. 6f,g). The NTS sh*Prlh* rats lost less weight and ate more food during cagrilintide treatment than controls, while semaglutide responses remained intact. Thus, Glu8.2 NTS *Calcr/Prlh* neurons play a crucial and specific role in cagrilintide action (Fig. 6h). Because sh*Prlh* treatment did not completely abolish cagrilintide’s effect, other neurotransmitters in the Glu8.2 NTS *Calcr/Prlh* neurons (for example, glutamate) or additional populations may also contribute to the sustained suppression of food intake and body weight.

## Discussion

We leveraged single-nucleus and spatial profiling techniques to generate a comprehensive spatially resolved caudal brainstem single-cell atlas and to identify DVC cell populations that mediate cagrilintide action. By analysing more than 530,400 cells across the mouse, rat and macaque brainstem, we identified 80 neuronal populations, 34 of which we predict reside within the rat DVC. Among these, we identified two conserved and cagrilintide-responsive neuronal populations, namely Glu4.2 *Calcr/Ramp3* neurons and Glu8.2 NTS *Calcr/Prlh* neurons. Cagrilintide activates and alters gene expression in Glu4.2 *Calcr/Ramp3* neurons in both species, whereas Glu8.2 NTS *Calcr/Prlh* neuron activation occurs specifically in rats. Given the stronger and more persistent food intake and weight loss responses to cagrilintide in rats than mice, this finding suggests the potential importance of Glu8.2 NTS *Calcr/Prlh* neurons in long-term cagrilintide-mediated weight loss. Indeed, activating AP *Calcr* neurons acutely decreases gastric emptying and food intake in rats but fails to suppress food intake and body weight long term. In contrast, knockdown of NTS *Prlh* attenuated cagrilintide-induced weight loss. Hence, the prolonged suppression of food intake and body weight by cagrilintide in rats likely requires PRLH signalling from Glu8.2 NTS *Calcr/Prlh* neurons. Furthermore, because semaglutide’s responses were unchanged in sh*Prlh* rats, these neurons likely play a role specific to amylin receptor agonists.

Although previous single-cell atlases of the AP and adjacent NTS (including ours) have been published^15,16,20^, the narrow dissection parameters in these studies incompletely covered the DVC. Additionally, previous studies neither examined the conservation of cell populations and receptor expression nor provided detailed spatial localization. The present study addresses these gaps, provides an easily accessible cross-species single-cell and spatial resource (see ‘Data availability’) and identifies DVC cells mediating amylin analogue action. Of eight neuronal populations expressing *Calcr* in at least one species, three were conserved across all three species. Thus, cross-species integration effectively prioritizes translationally relevant cell populations.

The Glu4.2 neuronal population from the present study contains a subpopulation (GLU4a) of the GLU4 AP *Glp1r* neuronal population from our previous AP-centred DVC atlas, as well as the GLU10 *Calcr/Ramp3* population. Although substantial *Calcr*/*Glp1r* co-expression exists in the mouse AP, *Calcr*-expressing and *Glp1r*-expressing Glu4.2 neurons mapped to different regions of the rat Glu4.2 UMAP plot, and rat AP *Calcr*^*Cre*^ neurons lacked GLP1R immunoreactivity. These findings suggest that related but functionally distinguishable subsets of Glu4.2 cells might exist. This segregation of the *Calcr*-expressing rat Glu4.2 cells from *Glp1r*-expressing rat Glu4.2 cells suggests that our AP *Calcr*^*Cre*^-based analysis is restricted to the *Calcr*-expressing subpopulation of Glu4.2 neurons.

Not all *Calcr*-expressing neurons responded transcriptionally to cagrilintide. For instance, we observed no change in the *Fos* regulon or overall gene expression in mouse or rat Glu8.0 NTS *Calcr* neurons by acute or subchronic cagrilintide; this may reflect relatively low *Calcr* expression in this neuron population (Fig. 2e). In the mouse, cagrilintide transcriptionally modulated Glu4.2 *Calcr/Ramp3* neurons only following acute treatment, but we observed no change in *Fos* regulon activity in these cells. The observed species differences in acute cagrilintide responses may reflect divergent pharmacokinetic or pharmacodynamic profiles, as prior studies have shown that neuronal activation (for example, FOS induction) peaks later in rats (~6 h) than in mice (~ 2 h) following s.c. administration, despite the use of doses considered equipotent at the receptor level^21^. Hence, we may have missed the optimal time point to detect *Fos* regulon activity in Glu8.0 *Calcr* and mouse Glu4.2 neurons by collecting tissue 4 h after cagrilintide treatment. Future studies examining a more detailed time course of cagrilintide responses would be informative.

Subchronic cagrilintide treatment in rats increased *Fos* regulon activity as well as gene expression in Glu8.2 *Calcr/Prlh* neurons. Indeed, *Prlh*, which is specific to Glu8.2 NTS *Calcr/Prlh* neurons, was the most significantly upregulated gene in the rat bulk RNA-seq analysis following subchronic cagrilintide treatment. These data implicate Glu8.2 NTS *Calcr/Prlh* neurons in cagrilintide action, consistent with their known roles in physiological control of food intake and body weight. Activation of mouse NTS *Calcr/Prlh* neurons reduces food intake and body weight and can override orexigenic signals from the hypothalamus^10,13^. The findings that NTS *Prlh* overexpression prevents diet-induced obesity, while silencing NTS *Calcr/Prlh* neurons or deleting *Prlh* from the NTS exacerbates DIO in mice, suggest that PRLH plays several roles in the control of body weight^13,22^. Loss-of-function mutations in the gene encoding GPR10 (the receptor for the *Prlh* peptide product, PrRP) are associated with severe obesity in humans and in mice^23^. Indeed, we found that NTS *Prlh* restrains feeding and body weight at baseline and mediates cagrilintide responses in the rat. Because some Glu8.2 neurons express *Glp1r*, semaglutide could potentially affect *Prlh* expression directly. However, NTS *Prlh* knockdown in rats had no effect on semaglutide-induced weight loss, suggesting that distinct neural systems mediate the majority of semaglutide action.

In contrast, activating AP *Calcr* neurons produced only short-term effects on feeding and failed to suppress food intake or body weight long term. These data suggest that Glu4.2 AP *Calcr/Ramp3* neurons are not sufficient to mediate cagrilintide-induced weight loss. Similarly, ablating AP *Calcr* neurons in rats did not alter cagrilintide action, although previous studies showed that AP ablation blunts amylin-stimulated NTS FOS accumulation and amylin-mediated suppression of feeding and body weight in rats^7,9^. Hence, *Calcr* neurons in or near the AP could theoretically contribute to amylin analogue action, even if they are not sufficient.

Our cross-species comparisons revealed that *Calcr* is specifically expressed in rat NTS Glu0.2 cells but not in mice or macaques. Because silencing rat NTS *Calcr* neurons was lethal while silencing mouse NTS *Calcr* neurons promotes weight gain on a high-fat diet (HFD)^13^, Glu0.2 neurons may play survival-critical roles, although the nature of these roles remains unclear. Unfortunately, the lethality effect prevented us from broadly manipulating NTS *Calcr* neurons, although we could specifically target Glu8.2 NTS *Calcr/Prlh* neurons via their unique *Prlh* expression.

Our findings provide a powerful and comprehensive new resource for exploring DVC cell populations, their conservation across species, and their roles in amylin analogue action. These data revealed, and we functionally confirmed, a crucial role for conserved Glu8.2 NTS *Calcr/Prlh* neurons in the anti-obesity effects of cagrilintide. Moreover, the inability to recruit Glu8.2 NTS *Calcr/Prlh* neurons may underlie the poor response to cagrilintide in mice. Rats may represent a better model for identifying and understanding anti-obesity medications for human use. Our findings also raise important questions, including why cagrilintide recruitment of Glu8.2 NTS *Calcr/Prlh* neurons is delayed in rats and how interspecies differences—such as the dominance of NTS *Glp1r/Prlh* neurons in macaques versus NTS *Calcr/Prlh* neurons in rodents—affect translational relevance.

## Methods

### Mouse in vivo cagrilintide treatment studies

#### Mice and rats

Twenty-week-old DIO (16 weeks on a HFD) male C57BL/6J mice were obtained from Charles River France. Upon arrival to the animal unit, DIO mice were single-housed with ad libitum access to 60% HFD (Research Diets, D12492) for 7 weeks. Lean Sprague–Dawley rats, defined as animals maintained on a low-fat diet (LFD; Lab Diet no. 5L0D) with ad libitum access, were single-housed in parallel with DIO rats. These lean animals provide a physiological baseline for comparison with the altered metabolic state of DIO rats. The animals were housed under a 12-h light–dark cycle in a temperature-controlled (21–23 °C) and humidity-controlled environment.

#### Randomization and dosing

Before study initiation, DIO mice were allocated into groups based on body weight. For the acute study, mice were dosed with a single s.c. injection of cagrilintide (10 nmol per kg body weight) or vehicle 0–1 h after onset of the light cycle. The mice were euthanized by decapitation under isoflurane anaesthesia 3–4 h after dosing. For the subchronic study, mice were dosed once a day with s.c. cagrilintide (10 nmol per kg body weight) or vehicle 3–4 h after onset of light for 7 days. One group of mice was weight-matched to mice dosed with cagrilintide by daily food restriction (Supplementary Fig. 1b). The day after the final dose, 3–4 h after onset of light, animals were euthanized by decapitation under isoflurane anaesthesia. The brains were excised followed by dissection of the DVC into RNAlater and stored at −80 °C until further processing.

#### Cagrilintide treatment of HFD-fed CalcR-cre^tdTomato^ mice (Supplementary Fig. 10)

Calcr-cre^tdTomato^ mice (Calcr^tm1.1(cre)Mgmj^ crossed with B6.Cg-Gt(ROSA)26Sor^tm14(CAG-tdTomato)Hze^ /J(007914); *n* = 24; 9 females, 15 males) were exposed to a 60% HFD (5.24 kcal per gram of food; SSNIFF Spezialdiäten, EF D12452) for 6 weeks. Before HFD exposure, mice were acclimatized to the housing conditions. They were pair-housed in individually ventilated cages furnished with a shelter and nesting material, in a temperature-controlled (21–22 °C) and humidity-controlled environment, under a 12–12 h light–dark cycle (lights off at 11:00). After 5 weeks of HFD exposure, six mice received daily s.c. injections of cagrilintide (10 nmol per kg body weight) for 7 days (subchronic treatment). At the end of the 6-week HFD period: the subchronic group received a final i.p. injection of cagrilintide and was euthanized 2 h later; six mice received an i.p. injection of vehicle and were euthanized after 2 h; six mice received an acute i.p. injection of cagrilintide and were euthanized after 2 h; and six mice received an acute i.p. injection of cagrilintide and were euthanized after 6 h (Supplementary Fig. 10). All animal experimental protocols in this study were approved by the University of Zurich Animal Protection Office and Ethics committee and the Cantonal Veterinary Office of the Canton of Zurich (licence nos. 106/2024 and 136/2021), and conform to Swiss Animal Protection guidelines and regulations (Swiss Animal Protection and Swiss Animal Act and Ordinance) and in accordance with the EU Directive on the protection of animals used for scientific purposes.

#### Perfusion of CalcR-cre^tdTomato^ mice

Mice were deeply anaesthetized (pentobarbital 100 mg per kg body weight, i.p; Kantonsapotheke Zurich) and transcardially perfused with cold 0.1 M phosphate buffer (PB) for 1 min followed by 4% paraformaldehyde (PFA) in PB for 1.5 min. Brains were removed and postfixed overnight in 4% of PFA and 30% sucrose in PB was used for cryoprotection for 24 h. Brains were frozen in hexane on dry ice and stored at −80 °C until sectioning. Brain AP and NTS sections were serially cut on a cryostat (CM3050S, Leica Biosystems), mounted on Superfrost Plus slides (Thermo Fisher Scientific) and cryoprotected in 50% PB, 30% ethylene glycol and 20%, and subsequently stored at −20 °C.

#### FOS immunostaining in the mouse

Blocking of the mouse brain sections was performed with 0.4% Triton–5% normal donkey serum (NDS) in PBS for 2 h at room temperature. AP/NTS sections were incubated at 4 °C for 48 h with rabbit anti-Fos antibody (1:500 dilution; Cell Signaling Technology, 2250, RRID: AB_2247211), and then after PBS washes, incubated with donkey anti-rabbit Alexa Fluor 488 (1:200 dilution; LuBio Science for Jackson ImmunoResearch, 711-545-1152) for 2 h at room temperature. After washing in PBS, the sections were counterstained with DAPI and coverslipped with Vectashield antifade Mounting Medium (Vectorlabs, H-1400).

#### GLP1R and CALCR protein immunostaining in the mouse

Brain AP and NTS sections from Calcr-cre^tdTomato^ and GLP1R-cre^tdTomato^ (Glp1r^tm1.1(cre)Lbrl/RcngJ^ crossed with B6.Cg-Gt(ROSA)26Sor^tm14(CAG-tdTomato)^) mice were used to stain for GLP1R protein and CALCR protein, respectively. Mice fasted for 12 h and were anaesthetized (pentobarbital 100 mg per kg body weight, i.p.) and perfused with 4% PFA. Brains were collected and processed as above.

GLP1R protein immunostaining in Calcr-cre^tdTomato^ mice was performed after an initial step of antigen retrieval with Tris-EDTA buffer (pH 9.0). AP/NTS sections were blocked in 0.3% Triton–3% normal goat serum in PBS for 2 h at room temperature. Sections were then incubated at 4 °C for 48 h with rabbit anti-GLP1R (1:500 dilution; anti-GLP1R antibody, EPR21819; Abcam, ab218532), and then after PBS washes, incubated with poly-horseradish peroxidase-conjugated 647 secondary antibodies for 1 h at room temperature, followed by a 7.5-min incubation with tyramide working solution (Thermo Fisher Scientific, B40926). CALCR protein immunostaining in GLP1R-cre^tdTomato^ mice was also performed after an initial step of antigen retrieval with Tris-EDTA buffer (pH 9.0). Sections were blocked in 0.3% Triton–3% NDS in PBS for 2 h at room temperature incubated at 4 °C for 48 h with rabbit anti-CALCR (1:2,000 dilution; anti-CALCR (EPR27455-47) Abcam, ab313335), and then after PBS washes, incubated with donkey anti-rabbit Alexa Fluor 488 (1:200 dilution; LuBio Science for Jackson ImmunoResearch, 711-545-1152) for 2 h at room temperature. After washing in PBS, the sections were counterstained with DAPI and coverslipped with Vectashield.

### Quantification and analysis of immunostaining in the mouse

Photomicrographs of all sections containing AP and NTS were taken at ×20 magnification using a Zeiss Axio Scan.Z1 slide scanner (Center for Microscopy and Image Analysis UZH). All images within each study were acquired using the same exposure time. Scanned images were quantified in QuPath (0.5.0). Three consecutive sections were counted and averaged for each area of interest. A polygon tool was used manually to determine the boundary of the region of interest of the brain section. A cell detection tool was used on the DAPI channel to detect every cell in the section by using a built-in cell segmentation algorithm. Two detection classifiers, for cFos and tdTomato signals, were created and the numbers of positive counted DAPI cells containing a cFos signal and a tdTomato signal were automatically counted within the region.

### Rat in vivo cagrilintide treatment studies

Twenty-two-week-old DIO (16 weeks on a HFD) male Spraque–Dawley rats were obtained from Charles River. Following arrival to the animal unit, DIO rats were single-housed with ad libitum access to 60% HFD (Research Diets, D12492) for 4 weeks. Lean rats were single-housed with ad libitum access to LFD. The rats were housed in a 12-h light–dark cycle in a temperature-controlled (21–23 °C) and humidity-controlled environment.

#### Randomization and dosing

Before study initiation, DIO rats were allocated into groups based on body weight. For the acute study, rats were dosed with a single s.c. injection of cagrilintide (3 nmol per kg body weight) or vehicle 0–1 h after onset of light. For the subchronic study, rats were dosed once a day with s.c. cagrilintide (3 nmol per kg body weight) or vehicle 3–4 h after onset of light. The vehicle group was weight-matched to rats dosed with cagrilintide by daily food restriction (Supplementary Fig. 1a). The day after the final dose, 3–4 h after onset of light, animals were euthanized by decapitation under pentobarbital anaesthesia. The brains were excised followed by dissection of the DVC into RNAlater and stored at −80 °C until further processing.

#### Non-human primates

DVC tissue from adult non-human primate rhesus macaques (three males, two females; 14–24 years old) was obtained from the Oregon National Primate Research Center (ONPRC). All animals were purpose-bred and born. All animal procedures were conducted in accordance with the guidelines of the Institutional Animal Care and Use Committee of the ONPRC. The ONPRC abides by the Animal Welfare Act and regulations enforced by the US Department of Agriculture. Macaques were deeply sedated with 20 mg per kg body weight ketamine, followed by 25 mg per kg body weight sodium pentobarbital, and then exsanguinated. Each brain was briefly flushed with heparinized saline, then removed from the skull and kept cold until dissection of the DVC area was performed. Tissue samples were rapidly frozen on dry ice and stored at −80 °C until further processing.

### Rat surgeries and analysis

#### Animals

All rats were housed in cages with Pure-o’Cel bedding and appropriate enrichment in the University of Michigan Vivarium. Rats were group-housed, except following surgery and during experimentation, during which time they were single-housed with extra enrichment. Food and water were provided ad libitum unless otherwise specified. Rats were maintained on a 12-h light–dark cycle in a temperature-controlled (21–23 °C) and humidity-controlled environment. The University of Michigan Animal Care and Use Committee (USA) approved procedures following the Association for the Assessment and Approval of Laboratory Animal Care and National Institutes of Health (NIH) guidelines, under protocols PRO00010555 and PRO00011225.

Male Sprague–Dawley rats for NTS *Prlh* knockdown were purchased from Charles River Labs and fed a HFD (Research Diets, D03082706) from the time of receipt. Previously described *Calcr*^*Cre*^ knock-in rats^12^ were bred in our colony at the University of Michigan and were fed normal chow (Purina Lab Diet 5L0D).

#### Stereotaxic surgeries and sh*Prlh* injections

Rats were handled for 3 days before surgeries. To perform DVC-targeted intracranial microinjections, rats were anaesthetized with isoflurane via an induction chamber at 3% and maintained under anaesthesia at 2–2.5%, delivered via face mask. Oxygen flow was delivered at 0.8 l min^−1^. Carprofen (s.c., 5 mg per kg body weight) injections were administered before surgery to assist with pain and inflammation. Rats’ heads were flexed at a 90° angle on a stereotaxic device. A vertical incision was made onto the skin through the three muscle layers in a foramen past the occipital bone. A horizontal incision was made on the meninges, revealing the obex located caudal from the fourth ventricle. Stereotaxic (model 942 Kopf with digital display console) surgeries were performed using a Hamilton syringe (5 μl syringe 800 series, 33-gauge small hub RN needle) for injections. Adult (30-week-old) *Calcr*^*Cre*^ rats of both sexes were used for injections of AAV8-hSyn-DIO-hM3Dq-mCherry or AAV8-hSynDIO-caCaspase3 and Sprague–Dawley rats were used for the injection of AAV8-hSyn-sh*Prlh*-GFP and AAV8-hSyn-shScrambled-GFP^24^ (all viruses were packaged at the University of Michigan Viral Vector Core) for AP injections, coordinates were zeroed in at the centre of the AP, and the virus was delivered unilaterally at a volume of 0.5 µl at coordinate *z* = −0.3 mm. For NTS injections, coordinates were zeroed in at the most ventral region of the AP, and bilateral injections of 0.2 μl per side using coordinates *x* = 0.5 mm and −0.5 mm, *y* = 0.5 mm and *z* = −0.6 mm. Postoperative care for all rats was conducted for 7–10 days. This included 3 days of carprofen treatment after surgery. Sutures were removed under isoflurane by day 10. For NTS sh*Prlh* rats and their controls, body weight and ad libitum food intake were monitored weekly for 5 weeks after surgery.

#### DREADD-mediated activation of rat AP *Calcr* neurons

*Calcr*^*AP-Dq*^ rats were tested no sooner than 21 days after surgery to allow for adequate viral expression. To examine short-term feeding and gastric emptying responses, we performed crossover studies in which the order of exposure to vehicle (0.9% sodium chloride, i.p.; Hospira) or CNO (Hello Bio; HB6149) was randomized. Each animal received a washout period of at least 5 days between studies. For short-term food intake, animals received i.p. injections 30 min before the onset of the dark cycle. Food was weighed at this time and again 2 h, 4 h, 6 h and 24 h later.

For gastric emptying, rats were fasted for 6 h during the early part of the light cycle. The fasted rats received i.p. injections 5.5 h after fasting. Thirty minutes later (6 h after the onset of the fast) the rats underwent an oral gavage of saline containing 0.1 mg per gram body weight acetaminophen. We collected blood samples from a tail snip 15 min after oral gavage. The blood was placed into a vessel containing EDTA and plasma was collected and frozen. Plasma acetaminophen concentrations were determined using the Acetaminophen L3K assay kit (Sekisui Diagnostics, 506-30). At the end of the study, the animals were supplied with food and water ad libitum.

For conditioned taste aversion assays, rats were habituated to injections and to a two-water bottle system for 3 days. The rats were water restricted for 24 h before conditioning to promote thirst. On the day of conditioning, rats were given two water bottles containing saccharin solution (0.15%, 240931, Sigma) at the onset of dark. After 2 h of exposure to the saccharin, rats were injected with a stimulus (vehicle, CNO (1 mg per kg body weight) or liraglutide (aversive control, 100 μg per kg body weight)). After injection, rats were returned to normal water bottles. The rats were again water restricted for 24 h before the test day. On test day, at the onset of dark, the rats were provided access to one bottle of water and one bottle of saccharin solution, and consumption of each was measured after 2 h. Saccharin preference was determined by comparing the volume of saccharin solution consumed to the volume of regular water consumed. At the end of the study, animals were provided ad libitum access to regular water.

For long-term food intake, we conducted a between-subjects design in which separate groups of control animals received CNO, cagrilintide or liraglutide and *Calcr*^*Cre-hM3Dq*^ rats received CNO. Each group underwent 8 days of injections. During the first 3 days, all rats received saline at the onset of the light cycle and 30 min before the onset of the dark cycle. The following 3 days, *Calcr*^*Cre-hM3Dq*^ and control *Calcr*^*Cre*^ rats received CNO (1 mg per kg body weight, i.p., twice a day); other control rats received saline at the onset of the light cycle and liraglutide (100 μg per kg body weight, i.p.) or cagrilintide (10 nmol per kg body weight, i.p.) 30 min before the onset of the dark cycle. Food and body weight were measured at the time of the evening injection. All animals received saline injections (i.p., twice a day) for the final 2 (recovery) days.

#### Cagrilintide treatment of AP caspase-treated and sh*Prlh* rats

For analysis of body weight and food intake changes under acute and long-term cagrilintide or semaglutide treatment, we conducted a crossover study in which order of vehicle (Novo Nordisk, −0000), cagrilintide (10 nmol per kg body weight, Novo Nordisk, −0833) or semaglutide (10 nmol per kg body weight) was randomized. Male rats received habituation injections i.p. of vehicle for 2 days before the beginning of the study. On day 1, animals were injected i.p. at the onset of the dark cycle, and food and body weight were measured. Food was again weighed at 2 h, 4 h and 16 h after injection. Subsequent injections were given daily for 7 days at the onset of the dark cycle. Food and body weight were measured at this time. Animals were allowed to recover to baseline body weight between studies.

#### Perfusions, immunohistochemistry and injection validation

Following the conclusion of in vivo studies, all animals (with the exception of sh*Prlh* rats and their controls, from which we dissected fresh brain tissue for qPCR analysis) were perfused for the analysis of virus localization (for example, mCherry-IR in hM3Dq-expressing rats), the presence of *Calcr* neurons, and in some cases for the analysis of FOS. A separate group of Calcr^AP-Dq^ rats was treated with CNO (1 mg per kg body weight, i.p., twice daily) for 3 days and then perfused. For perfusions, rats were overdosed with pentobarbital 200 mg per kg body weight (i.p.) and perfused 1.5 h (CNO or liraglutide) or 6 h (cagrilintide) after injection. Perfusions were carried out using a perfusion pump (Masterflex L/S Economy Variable-Speed Drive pump) with 1× PBS and 10% formaldehyde. The brain was extracted and placed in formaldehyde for 24 h, then transferred to 30% sucrose for 2 days. Brain tissue was sectioned (blade from Accu-Edge—Low Profile Microtome Blade, 4689) using a microtome (Leica) at −40 °C. Sections were 35-µm thick.

All Calcr^AP-Dq^ and Calcr^NTS-Dq^ brains were subjected to immunofluorescence staining for dsRed to visualize injection placement. Animals with inadequate transduction of the AP or NTS and/or misplaced injections were excluded from analysis. For immunostaining, brain tissue was incubated in primary antibody (rabbit anti-FOS (Cell Signaling Technology, 2250) or rabbit Anti-dsRed (Takara, 632496); all antibodies at a 1:1,000 dilution) at room temperature overnight followed by incubation with species-specific secondary Alexa Fluor anti-rabbit-488, anti-rabbit-568 (1:250 dilution; Invitrogen, A-11008). Sections were mounted on glass, superfrost, 75-mm × 25-mm microscope slides (Thomas Scientific, 1170Z86), coverslipped with 22-mm × 60-mm no. 1.5 microscope cover glass (Fisher Scientific, 12-541-026) and imaged on an Olympus BX53F microscope. Black and white images were pseudo-coloured using Cellsens Imaging software and ImageJ. Animals demonstrating off-target expression or poor transduction were excluded from analysis.

After the completion of studies, brains from sh*Prlh* rats and their controls were retrieved from deeply anaesthetized (isofluorane) rats and the NTS was dissected on ice. NTS tissue was frozen on dry ice and stored at −80 °C until the entire cohort of rats was collected. Total RNA was isolated with RNeasy Lipid Tissue Mini Kit (Qiagen, 74134) according to the manufacturer’s protocols. cDNA was prepared from 1 μg RNA using a High-Capacity RNA-to-cDNA Kit (Thermo Fisher, 4387406). qPCR was performed with the QuantStudio 3 Real-Time PCR machine (Applied Biosystems) using a Radiant Probe qPCR Kit (Alkali Scientific, QL4001) and Taqman gene assays (Thermo Fisher, 4331182) for *Prlh* (Rn00573653_m1) and *Grpr* (Rn01420745_m1). Relative mRNA expression was calculated with the delta-delta Ct method using *Gapdh* gene assay Rn01775763_g1 from Thermo Fisher as an endogenous control (4331182). NTS sh*Prlh* rats with *Prlh* expression similar to that of control rats were discarded from analysis.

### snRNA-seq

#### Generation of single-nucleus suspensions

Tissue was thawed for approximately 2 min on ice, then transferred into a 2-ml glass tissue douncer (Sigma-Aldrich, T2690) with 1 ml Nuclei EZ Lysis Buffer (Sigma-Aldrich, NUC101) and homogenized using pestle B. Following homogenization, the suspension was incubated for 5 min on ice, filtered through a pre-wetted 40-µm mini cell strainer (PluriSelect, 43-10040-40) into a 2 ml Protein LoBind tube (Eppendorf, 0030 108.132) and centrifuged for 10 min at 1,000*g* at 4 °C. The pellet was resuspended in 250 µl Nuclei EZ Lysis Buffer and 250 µl 83% OptiPrep (Sigma-Aldrich, D1556), gently layered on top of 500 µl 49% OptiPrep, in a 2 ml Protein LoBind tube, and centrifuged for 22 min at 14,000*g* at 4 °C. The isolated nuclei located at the bottom were resuspended in 500 µl Nuclei Buffer (PBS with 1% BSA (Sigma-Aldrich, A1595), 2 mM MgCl_2_ (Sigma-Aldrich, M1028), 40 U ml^−1^ Protector RNase inhibitor (Roche, 3335402001)) in a 1.5-ml Protein LoBind tube (Sarstedt, 72.706.605) and incubated on ice for 15 min, before centrifugation for 10 min at 1,000*g* at 4 °C. The final pellet was resuspended in 100 µl Nuclei Buffer for nuclei hashing.

#### Nuclei hashing for multiplexing

To the 100 µl single-nucleus suspension, 0.5 µg TotalSeq-A anti-nuclear hashtag with DNA-barcoded oligonucleotide (BioLegend) was added and incubated on ice for 30 min. A total of 1 ml Nuclei Buffer was added, and the suspension was centrifuged for 10 min at 1,000*g* at 4 °C. The pellet was resuspended in 200 µl Nuclei Buffer with 0.5 µg DAPI per µl (Chemometec, 910-3012).

#### Nuclei sorting

The hashed nuclei were sorted (SH800S, SONY) using the 70-µm Sorting Chip (Sony Biotechnologies, LE-C3207). The DAPI-positive nuclei were sorted into a 2-ml Protein LoBind tube with 18.8 µl RT Buffer B (10x Genomics, 2000165) for library preparation.

#### snRNA-seq library preparation

The volume of the sorted nuclei with RT Buffer was adjusted to 61.9 µl by adding Nuclei Buffer and processed using the Chromium Next GEM Single Cell 3’ Kit v3.1 (10x Genomics (1000268)), according to the manufacturer’s protocol. To generate a multiplexing Hashtag library, the BioLegend protocol (TotalSeq-A Antibodies and Cell Hashing with 10x Single Cell 3’ Reagent Kit v3.1 (single Index) Protocol) was followed from step II–III.

### snRNA-seq raw data processing

Raw reads were processed with Cell Ranger v7.1.0 (mkfastq, alignment to mm10, Rnor_6.0, or Mmul_10 genomes) using exonic and intronic reads. Ambient RNA was removed with CellBender (v0.2.2)^25^ (expected-cells = 7,000, total-droplets-included = 20,000, fpr = 0.1) run in cuda mode. For cell counting, an optimized framework was implemented using salmon v1.5.0 for indexing and alevin v1.5.0 in sketch mode for RNA and HTO alignment. Quantification was performed within the alevin-fry framework: valid barcodes were generated from the 10x Genomics February 2018 whitelist, corrected via the collate function, and counted using cr-like resolution strategy. Cell calling was performed in R on unspliced counts. Barcode ranks were calculated with DropletUtils v1.10.3 and a Hartigan’s dip test determined whether to apply a simple inflection cut-off (*P* ≥ 0.05) or fit a bimodal model using mclust v5.4.7 before recalculating inflection (*P* < 0.05). Overlapping RNA and HTO barcodes were imported into Seurat (v4.0.0)^26^, CLR-normalized and demultiplexed with HTODemux (positive quantile optimized across 0.50–0.99 in 0.01 increments). Intra-sample doublets were inferred using recoverDoublets from scDblFinder (v1.4.0)^27^. Filtered barcodes and CellBender gene counts were combined for downstream analysis.

### snRNA-seq data analysis

#### Initial processing

Each species was processed individually in Seurat. Two mouse batches (16 mice) were removed due to failed library preparation. Poor-quality cells were filtered out by unique molecular identifier (UMI) count and mitochondrial RNA content (mouse: UMI > 5 × 10^4^ or <500, mito > 2%; rat: UMI > 3 × 10^4^ or <500, mito > 2%; macaque: UMI > 5 × 10^4^ or <500, mito > 0.5%). Mouse and rat data were merged directly; macaque data were integrated using SCTransform, FindIntegrationAnchors and IntegrateData with canonical correlation analysis (CCA) on the top 3,000 variable genes. Principal component analysis (PCA) was run on the top 3,000 variable genes and initial clustering (resolution = 0.1, top 30 PCs) separated neurons from glia using known markers.

#### Cross-species integration

Neurons and glia from all three species, including cells from our published AP-centric atlas (reprocessed as below), were integrated after mapping rat and macaque genes to mouse orthologues (Ensembl BioMart). Integration used CCA on the top 3,000 variable genes, followed by PCA and clustering (resolution = 1, top 30 PCs). Low-count cells arising from orthologue mapping were removed (neurons > 2,000 counts; glia > 500). Low-quality clusters were identified by computing, per dataset and cluster, mean mitochondrial content and contamination marker expression (neurons: *Clic6*, *Mbp*; glia: *Slc32a1*, *Slc17a6*); clusters exceeding the 95th quantile in ≥3 of 4 datasets were discarded. For glia, putative doublet clusters co-expressing markers of distinct glial types (*Lkzf1*, *Slc38a5*, *Ntsr2*, *Myt1*, *Mbp*) above one-fourth of the maximum cluster mean in ≥3 datasets were also removed.

#### Neuronal clustering

Neurons were clustered (resolution = 1), yielding 55 major clusters split by neurotransmitter identity (*Slc32a1*, *Slc17a6*, *Chat*, *Slc6a4*). GABAergic (*n* = 19) and glutamatergic (*n* = 33) clusters were subclustered (resolution = 0.1), generating 84 clusters. Marker genes were identified with CELLEX (v1.2.2)^19^ (top 50 genes per cluster, >20% expression). Neighbour clusters with <10 separating markers were merged, yielding 80 final neuronal populations.

#### Differential expression

Pseudobulk expression matrices were constructed per animal and cell population (excluding combinations with <3 cells or populations with <2 replicates per group). DESeq2 (v1.30.1)^28^ Wald tests were performed on genes expressed in ≥10% of cells, with BH correction.

#### SCENIC analysis

SCENIC (pySCENIC v1.3.0)^29^ was run separately on mouse and rat neurons. Differential Fos regulon activity in *Calcr* populations was tested using linear mixed-effects models (treatment and *Calcr* expression as fixed effects, animal as random effect) with least-squares means *t*-tests and BH correction.

#### Reprocessing of the AP-centric DVC atlas

The published atlas was reprocessed with Cell Ranger v7.1.0 and CellBender using identical parameters. Poor-quality cells were removed (UMI > 1.5 × 10^4^ or <250, mito > 2%), merged, normalized and split into neurons and glia with recomputed PCA.

### Cross-species conservation analysis

Pairwise Pearson’s correlations of mean scaled expression for the 3,000 most variable genes were calculated across species to assess cell population conservation.

### Bulk RNA-seq

#### Library preparation

mRNA libraries were produced using the Illumina NovaSeq platform where paired-end reads (2 × 150 bp) were sequenced to a depth of approximately 2 × 10^7^ reads per sample.

#### Alignment and quantification

STAR v2.7.a3 was used for alignment and feature counting. The GRCm38 GENCODE vM23 genome and the corresponding rat genome were used for alignment of the mouse and rat data.

#### Outlier removal

Mouse and rat samples were subjected to vsd-transformation using DESeq2 v1.30.1 followed by PCA. Outliers were identified as being more than three standard deviations away from the mean PC1 or PC2.

#### Differential expression analysis

Differential expression analysis was performed with DESeq2 using the Wald test with the fraction of ribosomal RNA included as a covariate. To enable comparison between species, only orthologous genes identified in both species were included in the analysis. *P* values were corrected for multiple testing using the BH method (adjusting for the number of genes).

#### Logistic regression classification

Logistic regression classification with Lasso regularization was performed using the glmnet v4.1.1 R package. To increase speed, PCA was performed on the vsd-transformed bulk RNA-seq data and used as input for the regression analysis with the treatment group as the response variable and the principal components as the predictor variables.

To assess the performance of the regression classifier, the following steps were performed. The input data were split up into a training dataset comprising all samples except one and a test dataset comprising the held-out sample. A regression classifier was constructed on the training dataset. Leave-one-out cross-validation was applied to the training dataset for choosing the optimal regularization parameter *λ* through minimization of the cross-validation classification error using the ‘cv.glmnet’ function. The trained regression classifier was subsequently applied on the test dataset to compute a test classification error. These steps were repeated so that each sample was used to compute a test classification error of a regression classifier trained on the remaining samples. A mean classification error across all samples was subsequently calculated.

### Cell-type enrichment analysis

Differentially expressed genes identified from the bulk RNA-seq data were mapped to specific cell types by assessing the overlap with cell-type marker genes (ES_µ_ > 0) using a one-tailed Fisher’s exact test. *P* values were corrected for multiple testing using the BH method.

### Pathway analysis

Pathway analysis of the differentially expressed genes identified from the bulk RNA-seq was conducted by mapping gene symbols to Entrez IDs using the ‘mapIds’ function from the AnnotationDb R package. GO term enrichment was then assessed using the ‘enrichGO’ function from the ClusterProfiler^30^ R package, with the parameter ‘universe’ set to include all genes detected in the bulk RNA-seq data, ‘minGSSize’ set to 10, ‘maxGSSize’ set to 500 and ‘pAdjustMethod’ set to BH.

### In vivo data statistical analysis

Differences in body weight and food intake were assessed using a linear mixed-effects model with body weight or food intake as the response variable, treatment and day as predictor variables with interaction effects, and samples as random effects. Group contrasts were tested usings a least-squares means, two-tailed *t*-test. *P* values were corrected for multiple testing using the BH method.

### Spatial transcriptomics

#### Tissue preparation and staining

Fresh frozen brains from 8-week-old male chow-fed Sprague–Dawley rats (Janvier, France) were sectioned at a thickness of 12 µm to represent coronal and sagittal cuts of the DVC. No ethical permission was required from the Danish Animal Experiments Inspectorate because the animals were euthanized without undergoing experimental procedures. The slides were then processed with a proprietary multiplexed in situ hybridization protocol containing 100 genes (Resolve Biosciences) to yield a dataset of localized transcripts across the scanned brain areas. The sections were stained for RNA to better visualize neurons for cell segmentation. Five washes with PBS were followed by 2 h of biotinylated poly(T) (1:500 dilution; Z5261, Promega) in 0.4% Triton-100 at 41 °C. Another five washes with PBS were followed by 2 h of Streptavidin-647 (1:500 dilution; S21374, Invitrogen) in 0.4% Triton-100 at RT. Sections were then washed again five times in PBS before imaging.

#### Segmentation of spatial transcriptomics

To predict segmentation of the cell nuclei, spatial transcriptomics data were initially segmented using Cellpose (v.1)^31^. As an input, the DAPI and poly(T) stainings were stacked using ImageJ^32^. The cell segmentation and the spatial transcriptomics data (transcript location and identity) were then used as input to a custom pipeline that generates a spatial Seurat object (see our GitHub repository for implementation). The Seurat object contains a regular cell-by-gene matrix as well as the spatial information.

#### Cell population label transfer from single-cell to the spatial transcriptomics data

The spatial transcriptomics data underwent quality control using the transcript count and spatial cell size as input. Afterwards, spatial transcriptomics data were normalized using the SCTransform function. The snRNA-data were subsetted to all common genes between snRNA-seq data and spatial transcriptomics data. Cell population labels were then transferred from the single-cell data to the spatial transcriptomics data using the FindIntegrationAnchors and IntegrateData functions with ‘reduction’ set to CCA.

#### Cell population enrichment across DVC areas

To identify neuronal populations that were enriched in a specific DVC area (AP, NTS or DMV), a contingency matrix was constructed for each combination of neuronal population and DVC area. From the contingency matrix, the odds ratio was calculated, and statistical significance was evaluated using a Fisher’s exact test with an alpha of 0.05. Correction for multiple testing was performed using Bonferroni correction (adjusting for the number of cell populations × DVC area combinations).

### Multiplex in situ hybridization

Fluorescence in situ hybridization was performed on formalin-fixed paraffin-embedded brain blocks covering the DVC from rat, macaque and human material. The tissue blocks were sectioned at 5 μm and mounted onto Fisher SuperFrost Plus glass slides (Fisher Scientific). Multiplex FISH was performed using the RNAscope LS multiplex fluorescent reagent kit (Advanced Cell Diagnostics, Bio-Techne, 322800) together with iFluor546/iFlour594 (1:500 dilution; AAT Bioquest/VWR, AATB45035/AATB45025) and Opal690 Fluorophore Reagent pack detection (1:500 dilution; FP1497001KT, Akoya Biosciences) on a Leica BOND RX Fully Automated Research Stainer (Leica) according to the manufacturer’s instructions. Human and macaque tissue sections were pretreated for 30 min with HIER at 95 °C in ER2 (Leica) followed by a 15-min protease treatment and then hybridized with human-specific probes to detect mRNA transcripts for CALCR (483048), PRLH (526568-C2) and GLP1R (519828- C3; Advanced Cell Diagnostics, Bio-Techne). Rat tissue sections were pretreated for 15 min with HIER at 95 °C in ER2 (Leica) followed by a 15-min protease treatment and then hybridized with rat-specific probes to detect mRNA transcripts for CALCR (477798), PRLH (1301288-C2) and GLP1R (315228-C3; Advanced Cell Diagnostics, Bio-Techne). As a negative control, ACD 3-plex Multiplex Negative Control Mix (320878, ACD/Bio-Techne) was used. Slides were counterstained with DAPI and coverslipped with Prolong TM Gold Antifade mountant (Thermo Fisher Scientific). Fluorescent slide scans were acquired with an Olympus VS200 slide scanner (Olympus) using a ×20 (0.8 NA) air objective and 385/470;546/570;594/620;635/690 filter sets. Images were prepared with the Olympus OlyVIA software, and signal intensity levels were adjusted to match across staining/slides.

Image analyses were done using Halo 4.0 software (Indica Labs). Cell counts are reported as the percentage of GLP1R-positive and CALCR-positive cells per total PRLH-positive cells in the NTS. Image analysis was based on five rostral to caudal levels covering AP/NTS from bregma −13.68 mm to −14.28 mm in rats and bregma −33.3 mm to −34.65 mm in non-human primates. Image analysis in human samples covered one AP/NTS level per sample located between Obex +2.5 mm to −0.5 mm.

### Tissue sources for multiplex in situ hybridization

#### Human

Tissue blocks covering caudal medulla were obtained from the Edinburgh Brain Bank (*n* = 5, including both sexes (two males and three females), aged 50–85 years (BBN001.36970, BBN001.37233, BBN001.37268, BBN001.37340, BBN001.37380)) in collaboration with C. Smith (Edinburgh Brain Bank, University of Edinburgh), following UK and DK legal and ethical guidelines. Informed consent was obtained from all participants (no compensation involved). The remaining tissue from these blocks has been conserved for further analysis at Novo Nordisk, Denmark, and cannot be shared with other researchers upon request.

#### Non-human primate

Five levels of the caudal medulla were obtained at necropsy from purpose-bred cynomolgus monkeys (*Macaca fascicularis*; *n* = 4; three males and one female), originally used in a separate study conducted for research and safety testing purposes. The tissue utilized in this investigation represents secondary use and is not associated with therapeutic intervention. All animals were sourced in accordance with EU Directive 2010/63.

#### Rat

Two- and eighteen-month-old adult lean Sprague–Dawley rats (*n* = 12; six males and six females) were anaesthetized with isoflurane and cardiac perfusion fixation was performed using a peristaltic pump, starting with NaCl for 4 min and followed by neutral buffered formalin (VWR International) for 8 min. After overnight post-fixation in neutral buffered formalin, each brain was sliced into 2-mm slabs using a brain tissue matrix and processed to formalin-fixed paraffin-embedded blocks.

### Statistical analysis

Sample sizes for single-nucleus transcriptomics and DREADD experiments were determined based on prior experience with similar paradigms^12,13^. For cagrilintide treatment studies, DIO mice and rats were randomized into treatment groups based on body weight before study initiation. For DREADD experiments, the order of exposure to vehicle or CNO was randomized in a crossover design. Blinding was not performed, as downstream analyses relied on objective computational pipelines and experimental groups were defined by genetic modifications or viral transductions. Data distribution was assumed to be normal, but this was not formally tested. Differential expression analysis of snRNA-seq data was performed using DESeq2 (v1.30.1)^28^ Wald tests with BH correction. SCENIC^29^ regulon activity was tested using linear mixed-effects models with least-squares means *t*-tests and BH correction. Bulk RNA-seq differential expression was performed using DESeq2 Wald tests with the fraction of ribosomal RNA as a covariate and BH correction. Bulk RNA-seq outliers were identified as samples exceeding three standard deviations from the mean of PC1 or PC2. Logistic regression classification with Lasso regularization was performed using glmnet v4.1.1 with leave-one-out cross-validation to select the optimal regularization parameter. Cell-type enrichment was assessed using one-tailed Fisher’s exact tests with BH correction. Pathway analysis was performed using enrichGO from ClusterProfiler^30^ with BH correction. Cross-species conservation of cell population gene expression was assessed using pairwise Pearson’s correlations. Cell population enrichment across DVC areas was evaluated using Fisher’s exact tests with Bonferroni correction. In vivo body weight and food intake data were assessed using linear mixed-effects models with treatment and day as fixed effects and animal as a random effect; group contrasts were tested using least-squares means two-tailed *t*-tests with BH correction. All statistical details, including test statistics and exact *P* values, are reported in the figure legends and supplementary tables.

### Reporting summary

Further information on research design is available in the Nature Portfolio Reporting Summary linked to this article.

## Supplementary information

Supplementary Information Supplementary Figs. 1–16, and legends for Supplementary Tables 1–12.

Reporting Summary

Supplementary Table 1 Number of animals per treatment for each species.

Supplementary Table 2 Number of neuronal and glial cells per treatment for each species.

Supplementary Table 3 Tabulated overview of all single-nucleus sequencing metadata for each species.

Supplementary Table 4 List of 100 genes targeted in the spatial transcriptomics imaging of the rat DVC. Each item includes the catalogue number of the manufacturer for the probe, the species, the ENSEMBL ID and the canonical gene name. Two items (*Lepr* and *Foxj1*) are custom probes.

Supplementary Table 5 Spatial enrichment of each neuronal cell type to regions in the DVC (AP, NTS or DMV) denominated by the −log_10_ *P* value for the respective cell type to be enriched in the region. Note that Sero0.0 has been excluded from the report because it was found to map outside the anatomical area that was covered by spatial transcriptomics and is therefore false positive in this analysis.

Supplementary Table 6 Genetic enrichment (ES_µ_) values for each cell type and species for *Calcr*. Values above the threshold (>0.8) are highlighted.

Supplementary Table 7 Tabulated results of quantified immunohistochemistry sections of the mouse DVC. Results are split for the AP and NTS and contain the absolute number and percentage of counted cells for each animal. Mice were labelled with Glp1r-Cre crossed to a tdTomato Cre-reporter line and stained for *Calcr* expression to quantify co-expression.

Supplementary Table 8 Tabulated results of quantified immunohistochemistry sections of the mouse DVC. Results are split for the AP and NTS and contain the absolute number and percentage of counted cells for each animal. Mice were labelled with Calcr-Cre crossed to a tdTomato Cre-reporter line and stained for Glp1r expression to quantify co-expression.

Supplementary Table 9 Differential gene expression analysis results of rat bulk RNA-seq across all treatments. *P* values were adjusted for multiple testing using BH FDR correction.

Supplementary Table 10 GO enrichment analysis of differentially expressed genes in rat bulk RNA-seq across all treatments. Enrichment was carried out only on the significantly regulated genes (*P*_adj_ < 0.05) using all tested genes as background. GO terms with BH-adjusted *P* values < 0.05 are reported.

Supplementary Table 11 Cell population-specific differential gene expression analysis results of rat and mice across all available treatment. *P* values were adjusted separately using BH FDR correction for each of the 20 species treatment cell-type contrasts. Genes with an adjusted *P* value < 0.05 were concatenated and shown.

Supplementary Table 12 Cell population-specific differential gene expression analysis results of DIO mice versus chow mice. *P* values were adjusted separately for each cell type using BH FDR correction. Genes with an adjusted *P* value < 0.05 were concatenated and shown.

## Source data

Source Data Fig. 1 Cell-type-proportion source data.

Source Data Fig. 3 Spatial bregma and dataset mapping source data.

Source Data Fig. 4 Regulon analysis and generalized linear model source data.

Source Data Fig. 5 Statistical source data for mouse experiments.

Source Data Fig. 6 Statistical source data for mouse experiments.

## Data Availability

snRNA-seq (E-MTAB-16929) and bulk RNA-seq (E-MTAB-16870) data are available in EMBL-EBI BioStudies. Spatial transcriptomics data are available at Zenodo via 10.5281/zenodo.19368177 (ref. ^33^). Rat spatial data are available at https://cbmr-rmpp.shinyapps.io/spatial_dvc_app/. Source data are provided with this paper.

## Code availability

All code used to analyse the data is available at https://github.com/perslab/ludwig-coester-gordian-2025/.
