# Supplementary material for: A cross-species atlas of the dorsal vagal complex reveals neural mediators of the effects of cagrilintide on energy balance
Source: Nat Metab. 2026 Jun 8;8(6):1350–67. doi: 10.1038/s42255-026-01539-3 (PMC13303089; doi:10.1038/s42255-026-01539-3)
Supplement: Supplementary file 1 — Supplementary Figs. 1–16, and legends for Supplementary Tables 1–12. [file 42255_2026_1539_MOESM1_ESM.pdf]

nature metabolism

Supplementary information https://doi.org/10.1038/s42255-026-01539-3

A cross-species atlas of the dorsal vagal complex reveals neural mediators of the effects of cagrilintide on energy balance

In the format provided by the authors and unedited

Supplementary Figures

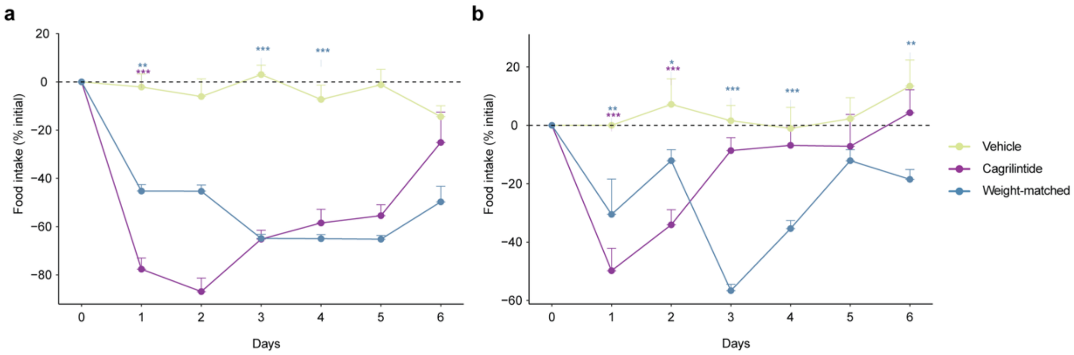

Supplementary Figure 1. a,b, Daily food intake relative to the initial value in rats (**a**) and mice (**b**) following cagrilintide or vehicle administration and pair-fed controls (**a** = 10 rats; **b** = 7-8 mice). Values are the mean ± s.e.m. **P* < 0.05, ***P* < 0.01, ****P* < 0.001 versus vehicle.

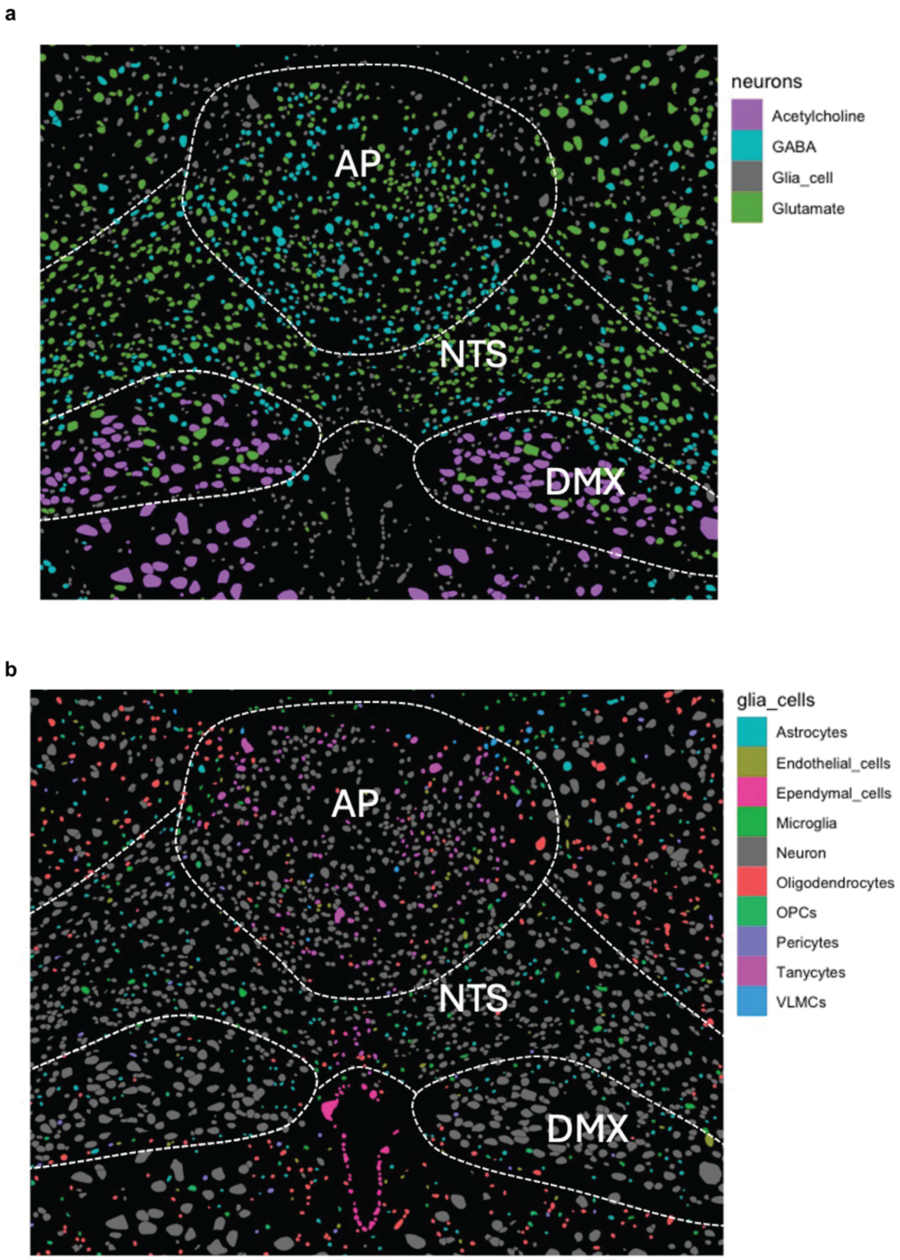

Supplementary Figure 2. a, Segmented neurons plotted in the dorsal vagal complex at Bregma -14.16 in the rat brain, colored by major neurotransmitter classes (Acetylcholine, Chat; Gamma-aminobutyric acid, GABA; Glutamate, Glu). **b,** Identical plot with glial cells annotated.

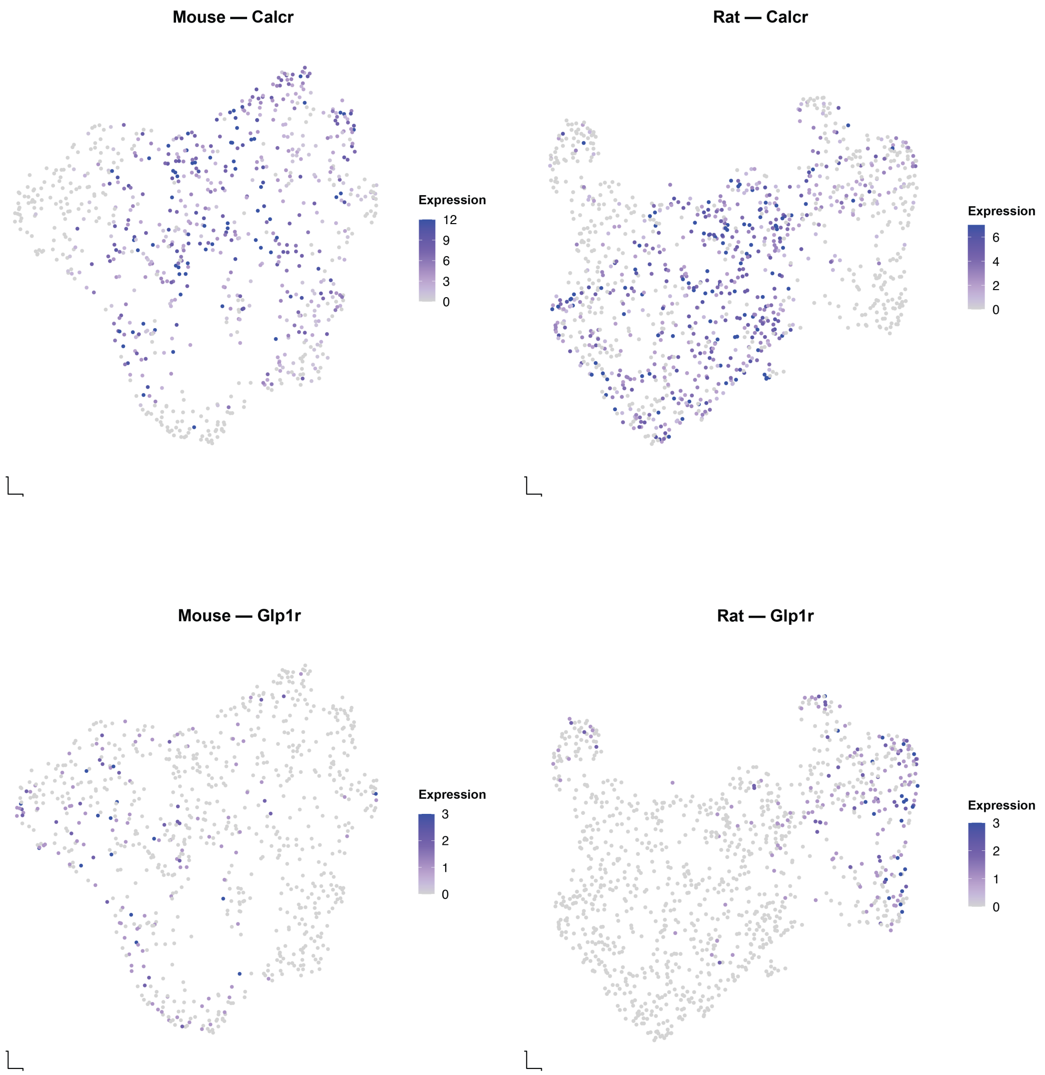

Supplementary Figure 3. UMAP feature plot of Glu4.2 neurons with *Calcr* (top panels) and *Glp1r* (Bottom panels) in mouse (left panels) and rat (right panels). Shown are raw expression values

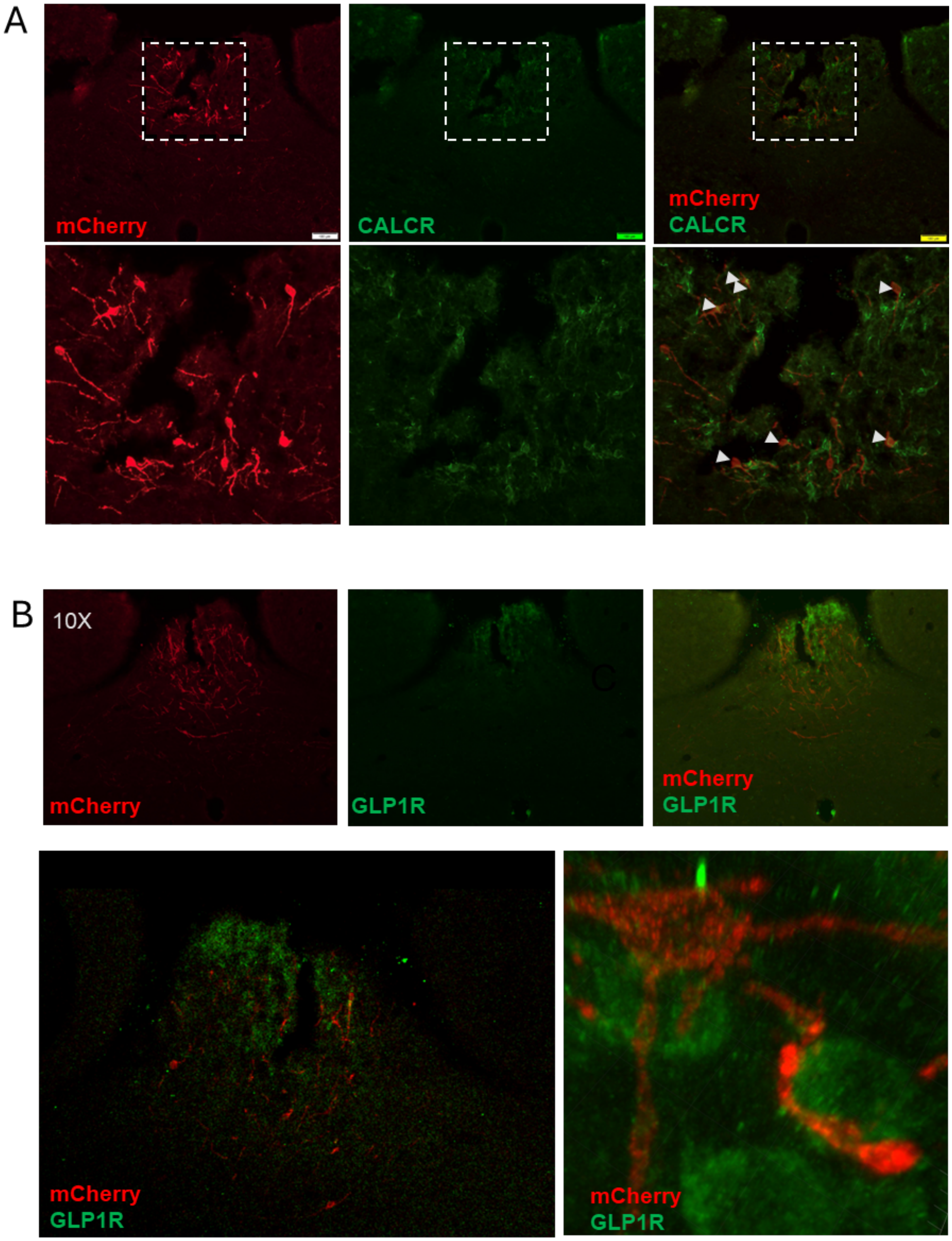

Supplementary Figure 4. Immunostaining for mCherry, CALCR and GLP1R in $Calcr^{AP\text{-}Dq}$ rats. **a,** Representative images (from >5 brains) of mCherry-IR (red, left) and CALCR-IR (green, middle) in the DVC of $Calcr^{AP\text{-}Dq}$ rats. Merged images are shown on the right. Images in the lower panels are digital

zooms of boxed areas in top panels. Arrowheads indicate examples of colocalized cells. Scale bar=200 um. **b,** Representative images (from >5 brains) of mCherry-IR (red, left) and GLP1-R (green, middle) in the DVC of Calcr^{AP-Dq} rats. Merged images are shown on the right. Lower panels represent confocal images- left panel is a z-stack; right panel demonstrates lack of colocalization between mCherry-IR and GLP1R-IR cells that overlap on epifluorescence images.

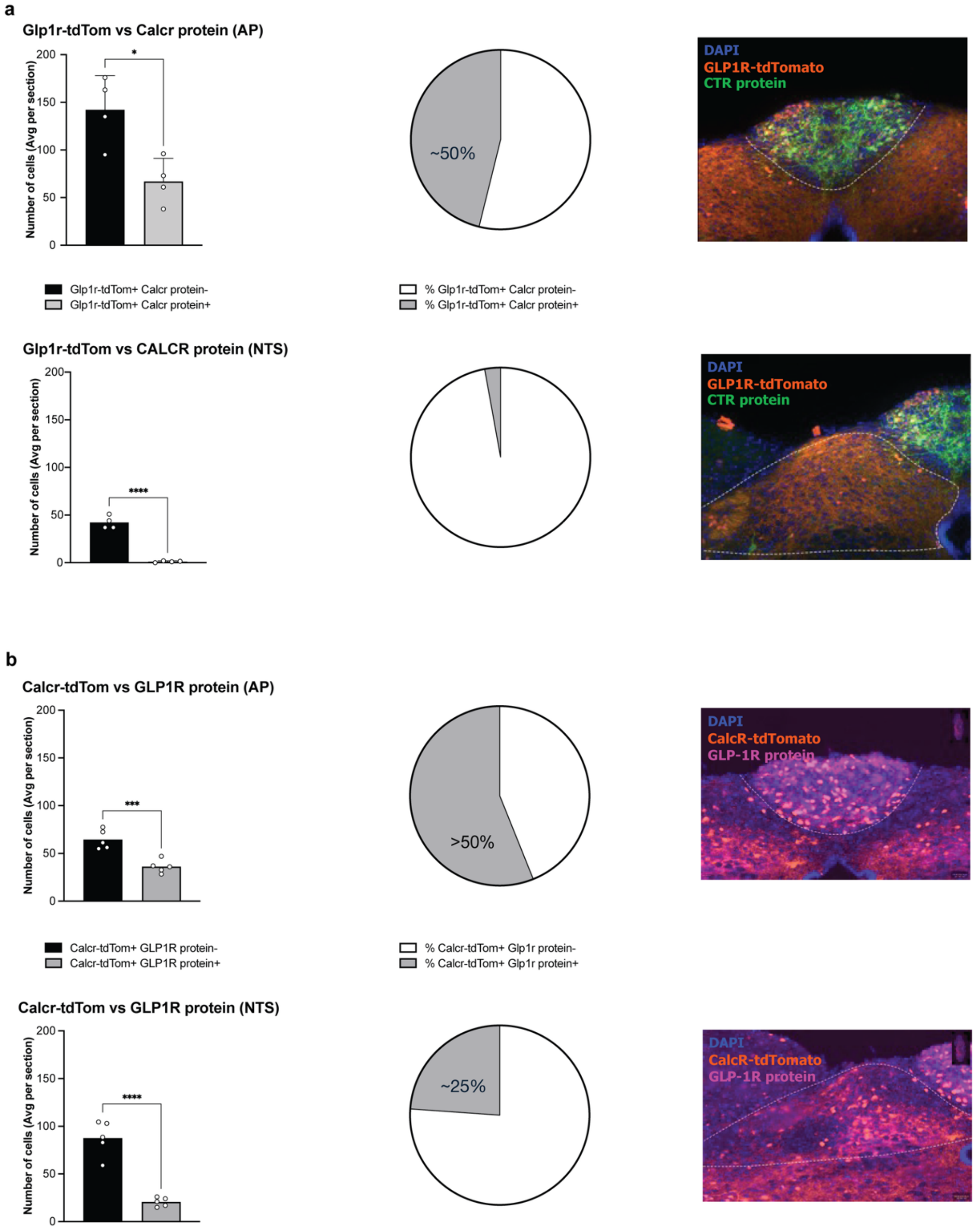

Supplementary Figure 5. a, Immunostaining for CALCR or GLP1R and tdTomato in AP and NTS of GLP1R-Cre x tdTomato mice. **b,** CALCR and GLP1R protein colocalisation in AP and NTS CalcR-Cre x tdTomato (a = 4 mice; b = 5 mice). Data are expressed as number of cells or as percent of tdTomato labelled cells and represented as mean +/- S.D. *$p<0.05$, ***$p<0.001$, ****$p<0.0001$.

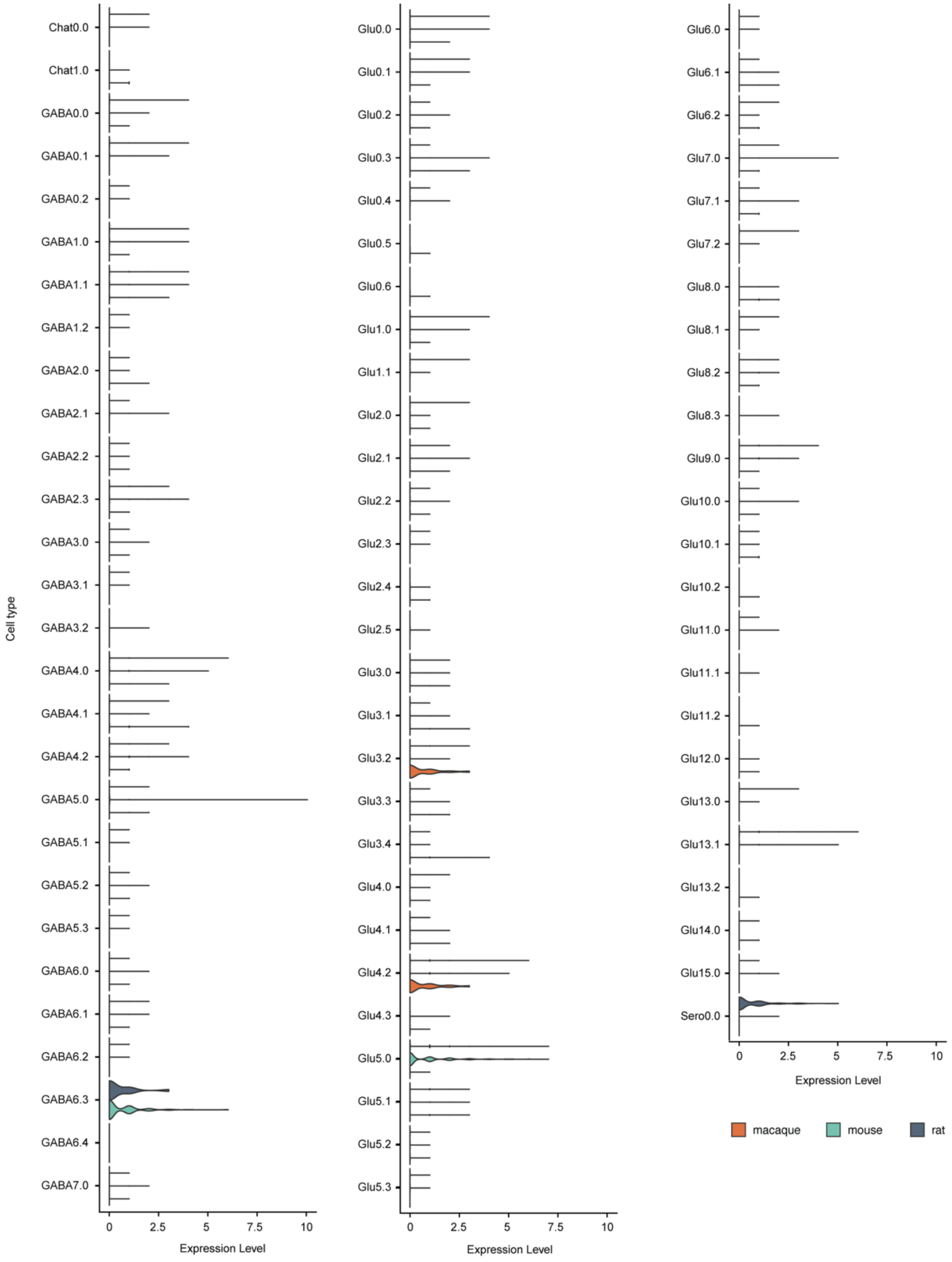

Supplementary Figure 6. Violin plot showing expression of *Glp1r* across all DVC neuronal cell populations in mouse, rat, and macaque.

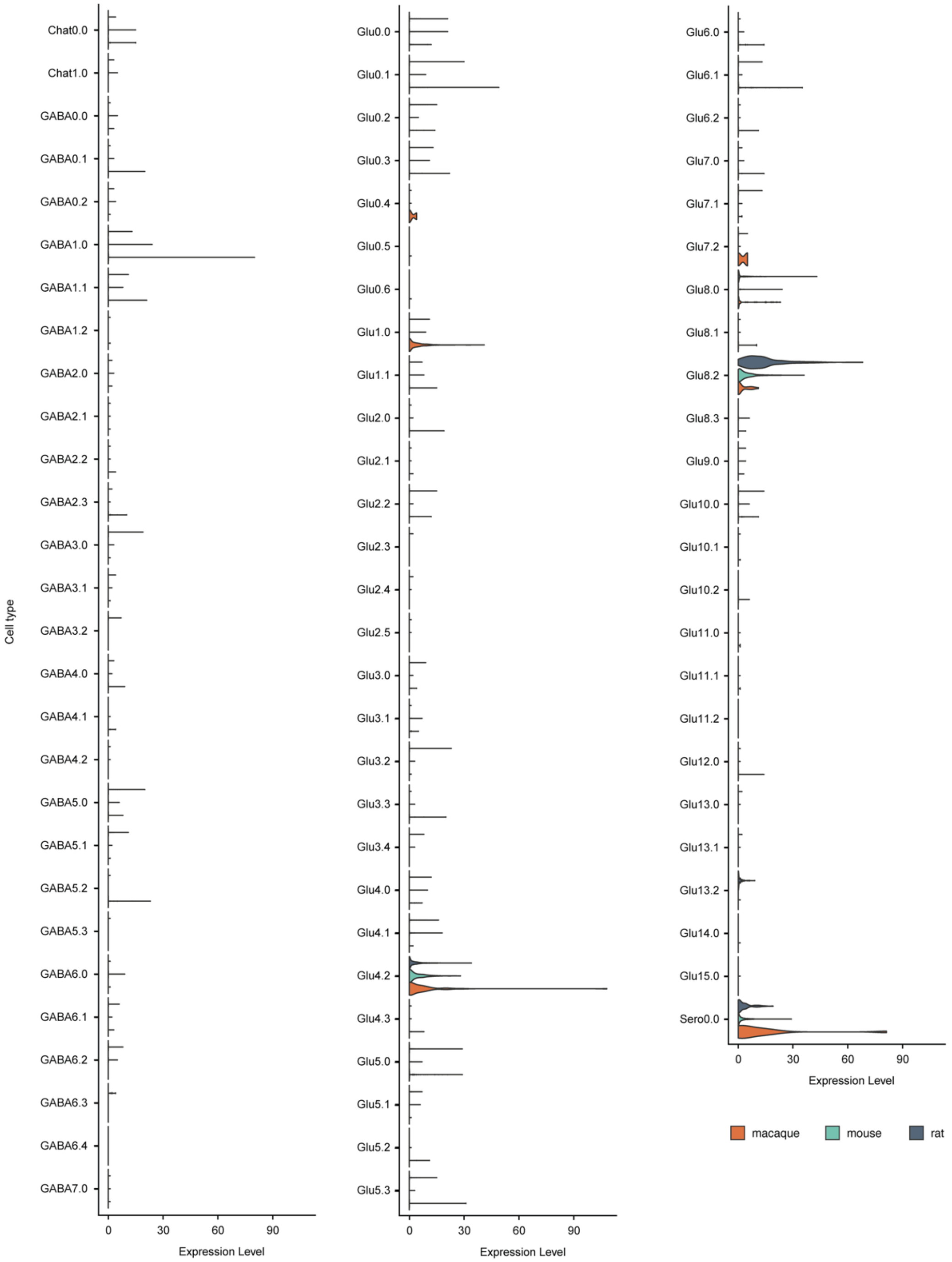

Supplementary Figure 7. Violin plot showing expression of *Calcr* across all DVC neuronal cell populations in mouse, rat, and macaque.

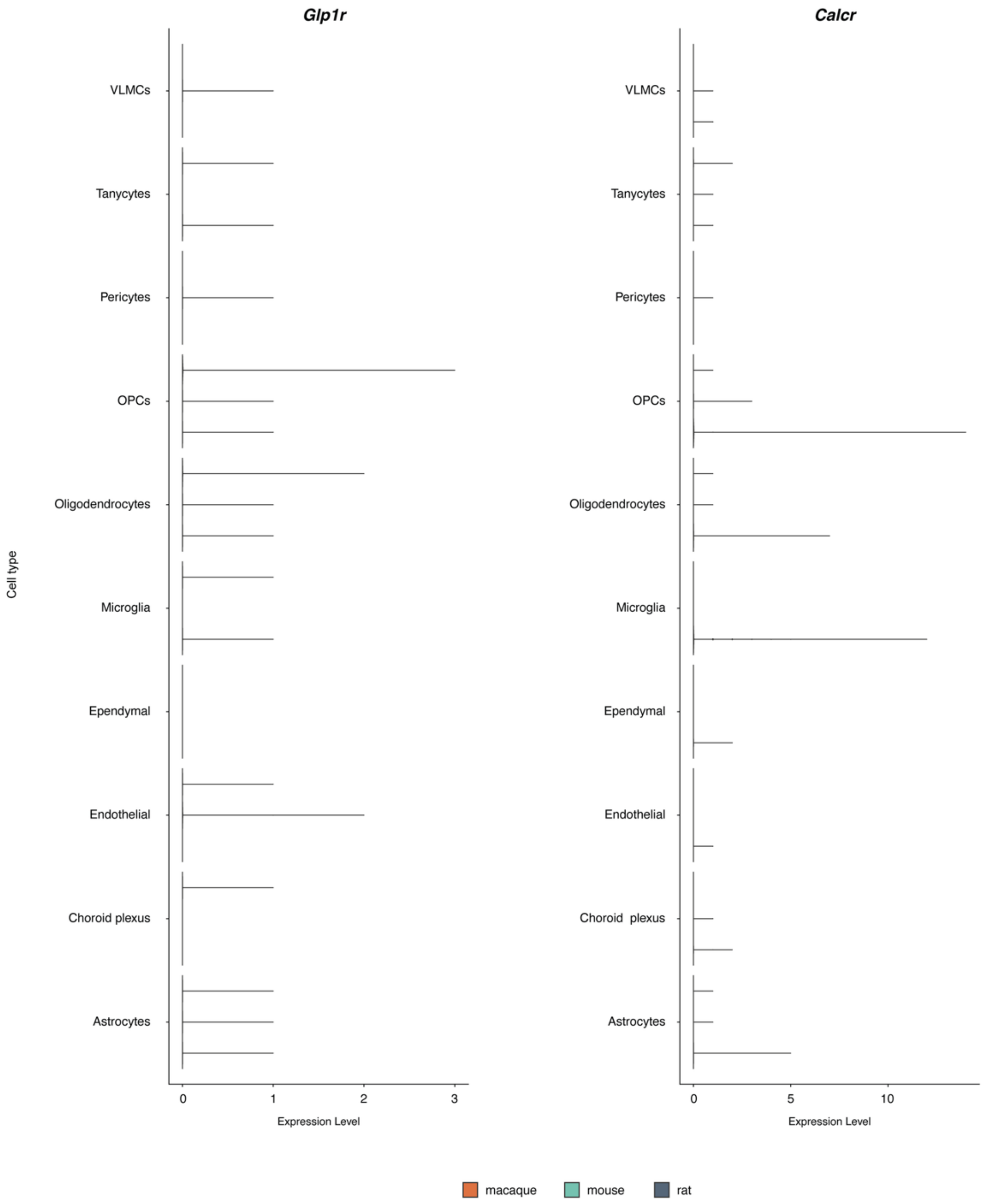

Supplementary Figure 8. Violin plot showing expression of *Glp1r* and *Calcr* across all DVC glia cell populations in mouse, rat, and macaque.

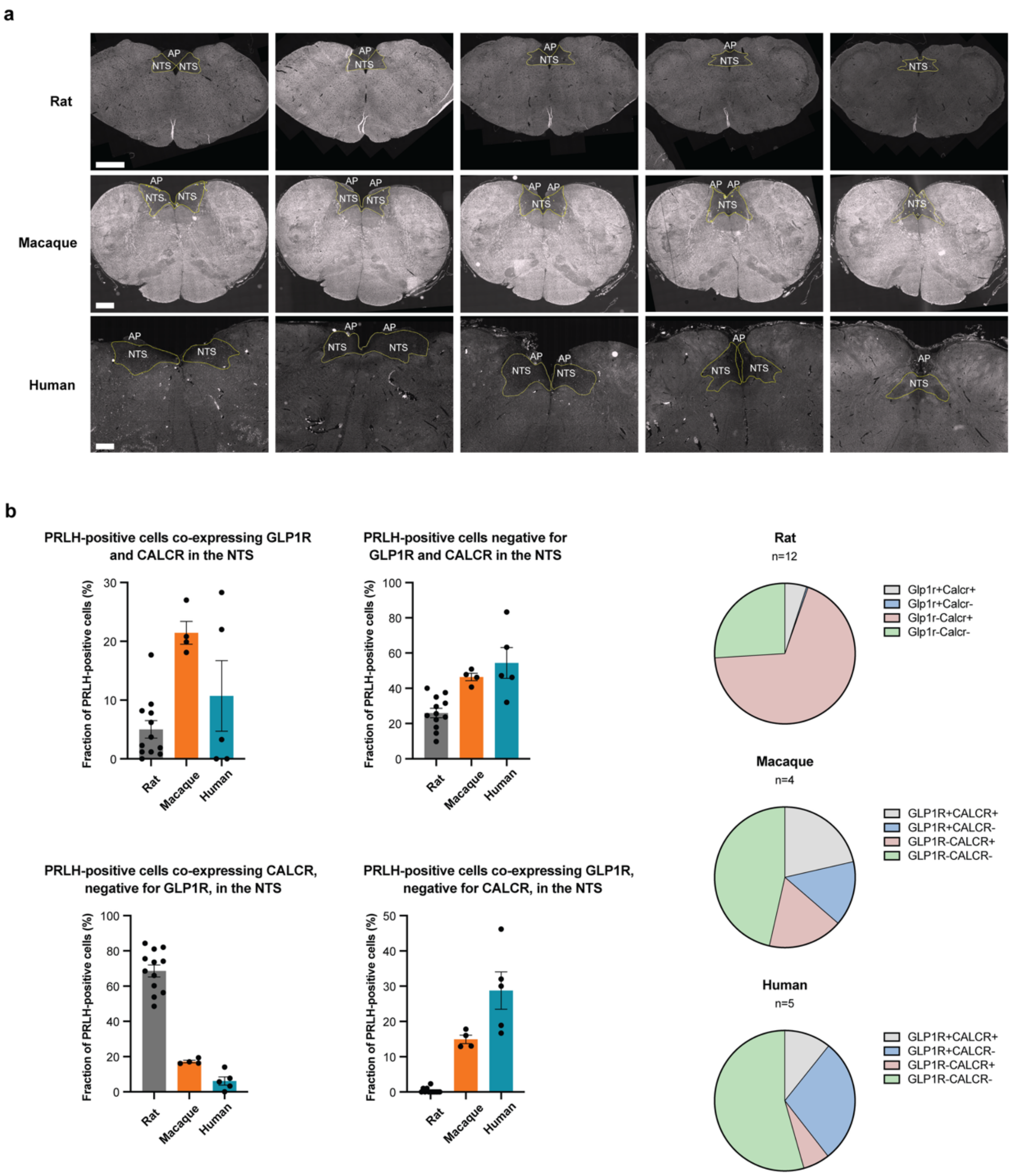

Supplementary Figure 9. a, Representative images of five rostral to caudal levels covering AP/NTS from Bregma -13.68 mm to -14.28 mm for rat and Bregma -33.3 mm to -34.65 mm for NHP. Human samples cover one AP/NTS level per individual located between Obex +2.5 mm to -0.5 mm. Scale bars are equal to 1 mm. **b,** Quantification of RNAscope ISH for *GLP1R*, *CALCR* and *PRLH* shown in Figure 3e. *PRLH*-positive cells were counted and assessed for co-expression with *CALCR* and *GLP1R* in rats (n=12), macaques (n=4) and humans (n=5). Fractions of *PRLH+/CALCR+* and *PRLH+/GLP1R+* in each species are shown as bar graphs (Data+/-SEM). Relative abundance of each combination is shown as pie graphs. Abbreviations: AP, area postrema; NTS, nucleus of the solitary tract.

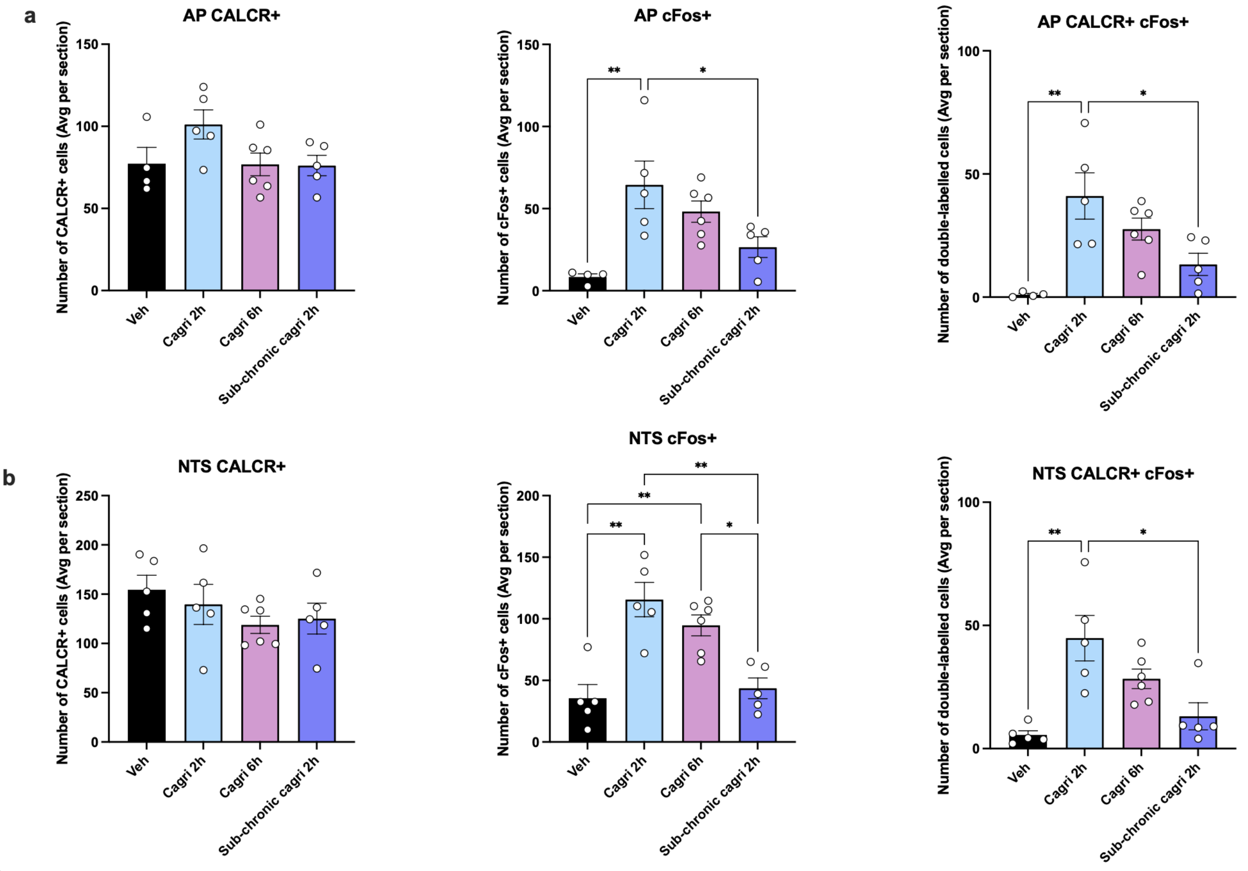

Supplementary Figure 10. Quantification of Calcr-tdTomato- and FOS-immunoreactivity in AP (**a**) and NTS (**b**) of HFD-fed CalcR-Cre tdTomato mice treated acutely with cagrilintide (sacrificed after 2h and 6h) or subchronically for 7 days (sacrificed 2h after the last injection) (mice = 5 per group). Data are expressed as number of cells represented as mean +/- SD. *p<0.05, **p<0.01.

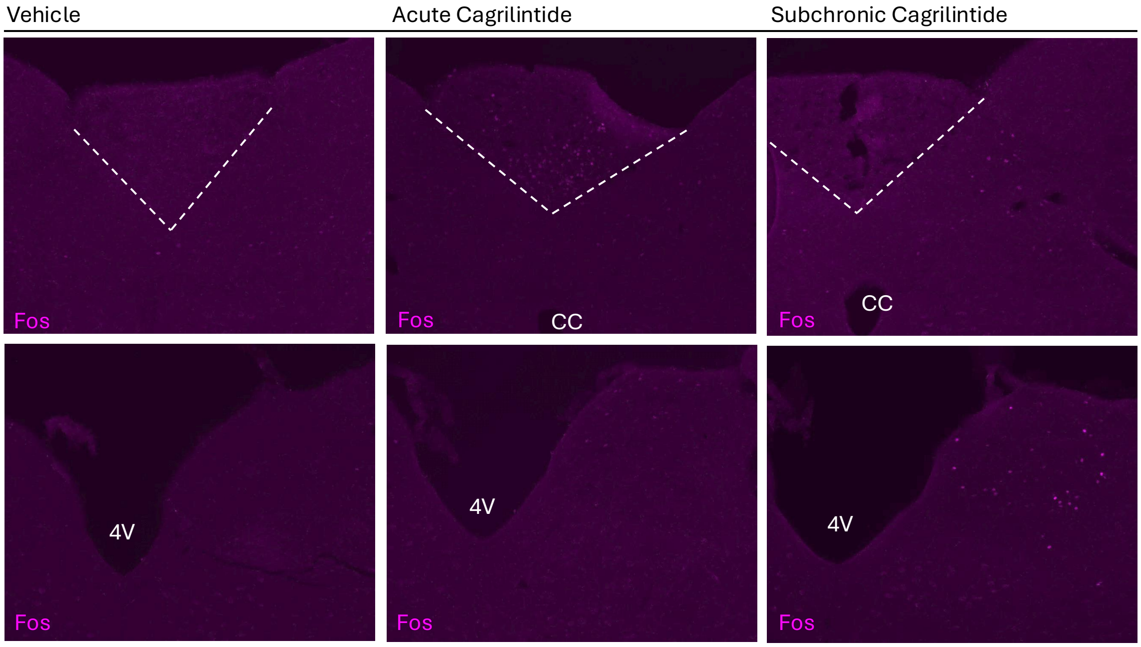

Supplementary Figure 11. Representative images showing FOS-IR in the DVC of rats treated with vehicle, acute cagrilintide (4 hours), or subchronic cagrilintide (7 days).

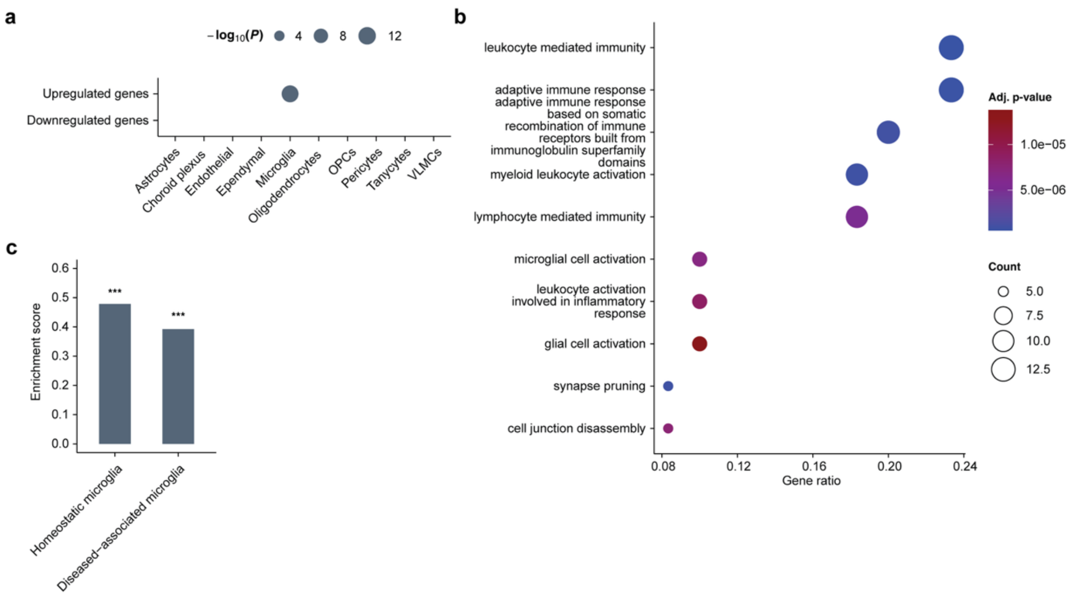

Supplementary Figure 12. Glial cell type response to cagrilintide. **a**, Glial cell type enrichment of bulk RNA-seq differentially expressed genes induced by subchronic cagrilintide treatment in rats. **b**, Top 10 most enriched gene ontology terms for the upregulated genes in panel a, colored by BH-adjusted *P*-value. **c**, Gene set enrichment analysis of markers for homeostatic and disease-associated microglia in bulk RNA-seq of subchronically cagrilintide-treated vs. weight-matched control rats. *** $P<0.001$.

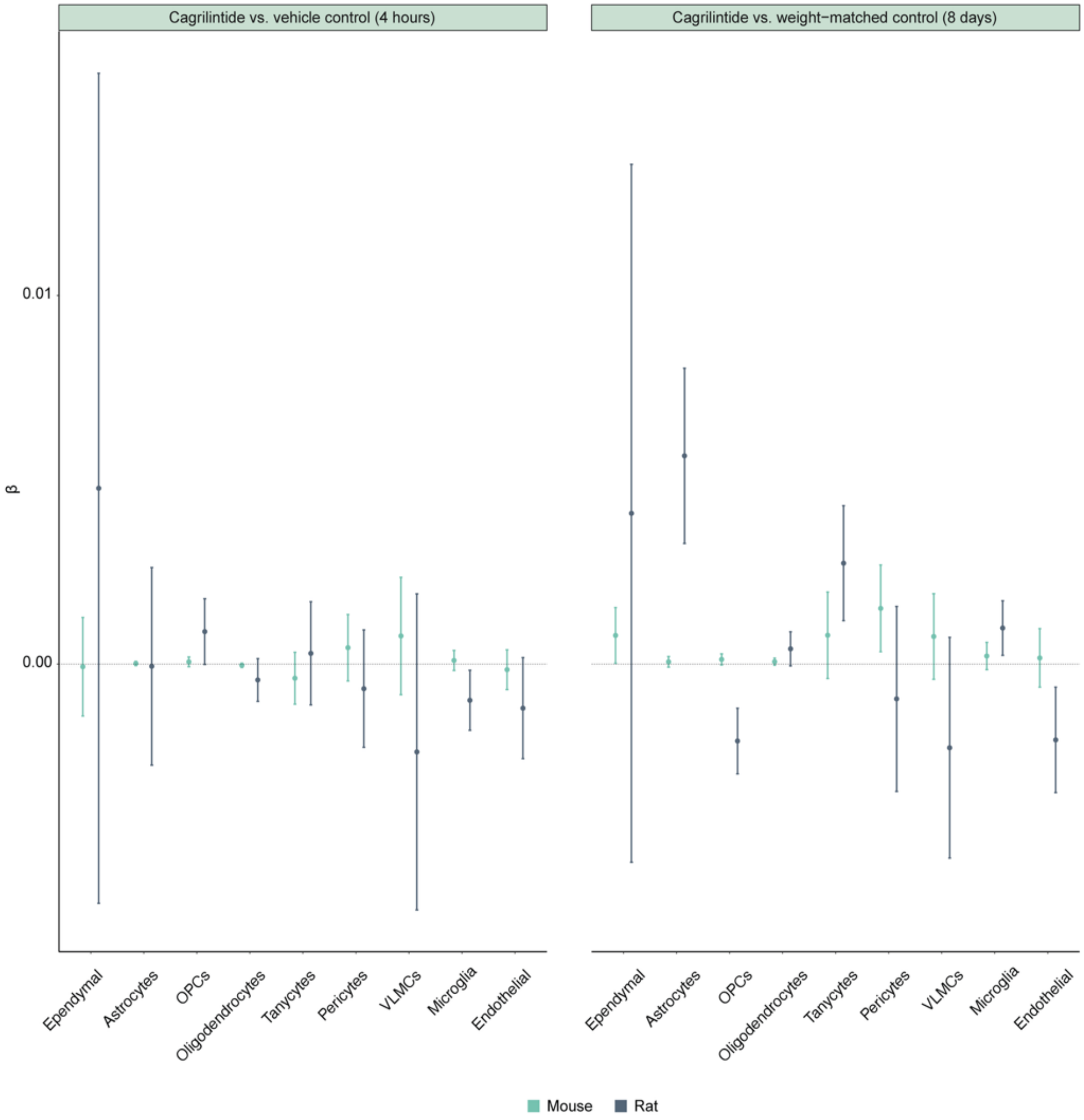

Supplementary Figure 13. Change in activity of Fos-regulated genes in glia of mice and rats between exposure to cagrilintide and vehicle administration after single-nucleus RNA-seq of the DVC. Values are the mean estimate ± SE. Linear mixed-effects model with sample included as covariates (n=6 mice per group, 10-11 rats per group), followed by a BH-adjusted two-tailed least-squares means t-test.

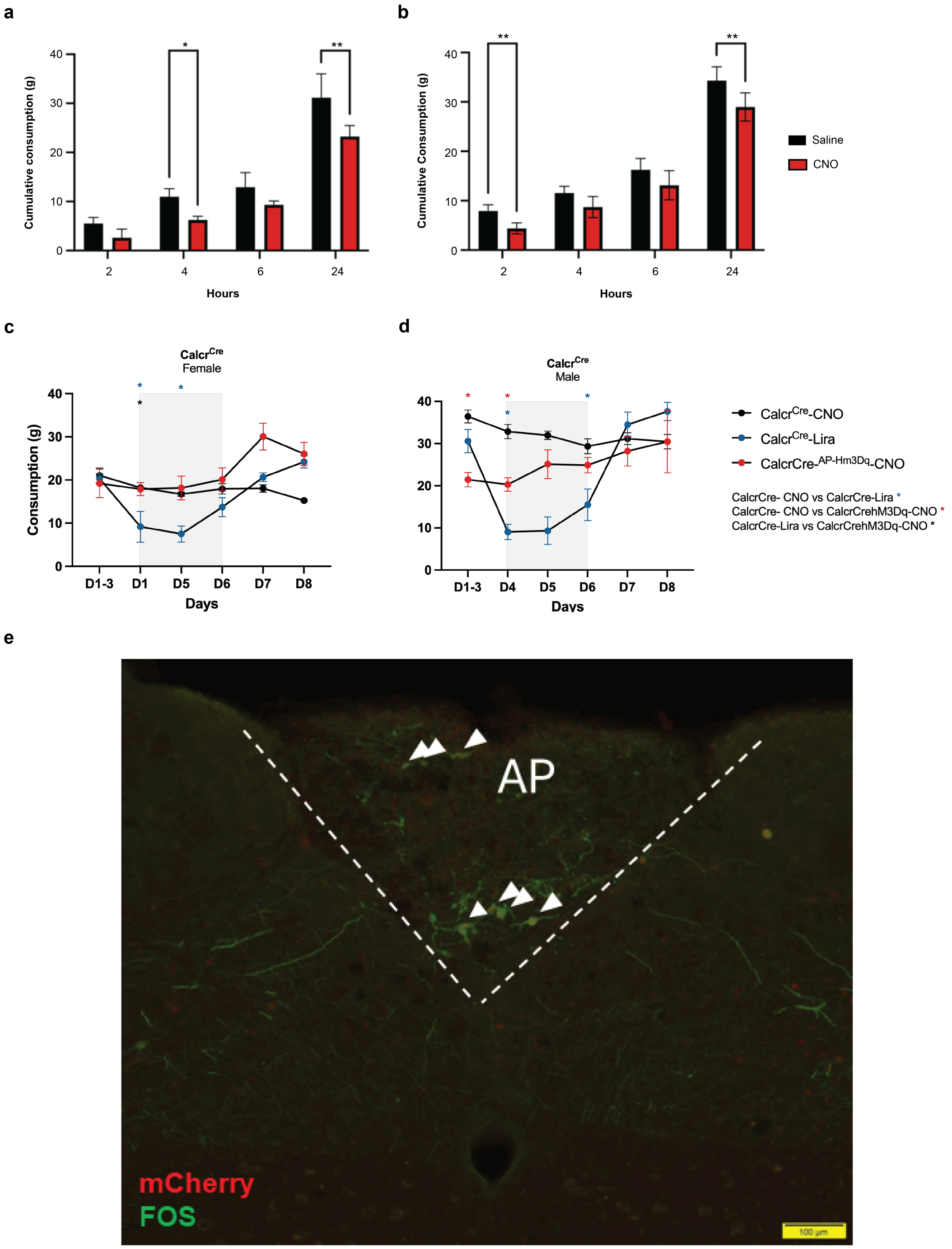

Supplementary Figure 14. **a**,**b**, Food consumption in grams, by sex (left panel- female, right panel- male), for animals in Figure 5d. **c**,**d**, Long-term consumption in grams by sex (left panel- female, right panel- male) for CalcrCre-CNO, CalcrCre-Lira, and CalcrCre-AP-hM3Dq-CNO animals. Linear effects model A linear effects model analysis, * p<0.05. **e**, representative image showing colocalization of mCherry-IR (green) and FOS-IR (red) in $Calcr^{AP\text{-}Dq}$ animal following 72 hours of repeated CNO dosing. AP is shown and AP/NTS

border is indicated with dashed white lines. Arrowheads indicate examples of mCherry-IR cells containing FOS-IR. Scale bar=200 um.

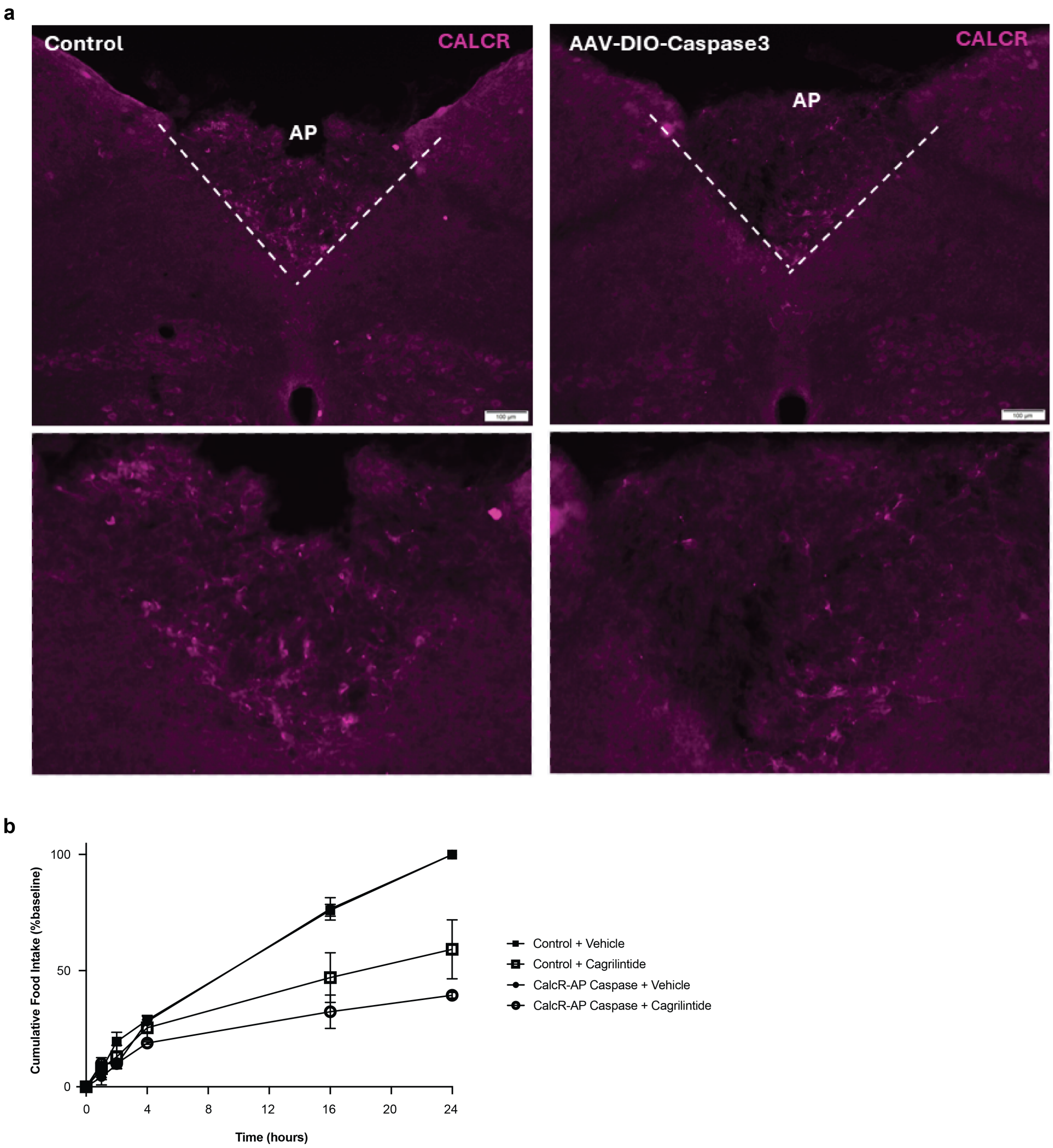

Supplementary Figure 15. Effect of ablating AP Calcr neurons on cagrilintide action. **a**, representative images of CALCR-IR control (left) and AAV-DIO-Caspase3-injected (right) Calcr-Cre animals. AP is labelled and AP/NTS border is indicated by dashed while lines. Scale bar= 200 um. Lower panels show digital zooms of the boxed areas in the upper panels. **b**, Cumulative food intake over 24 hours for control (n=4) and Calcr-AP Caspase (n=2) animals treated with cagrilintide (10 nmol/kg, sc) at the onset of the dark cycle. No significant differences by group.

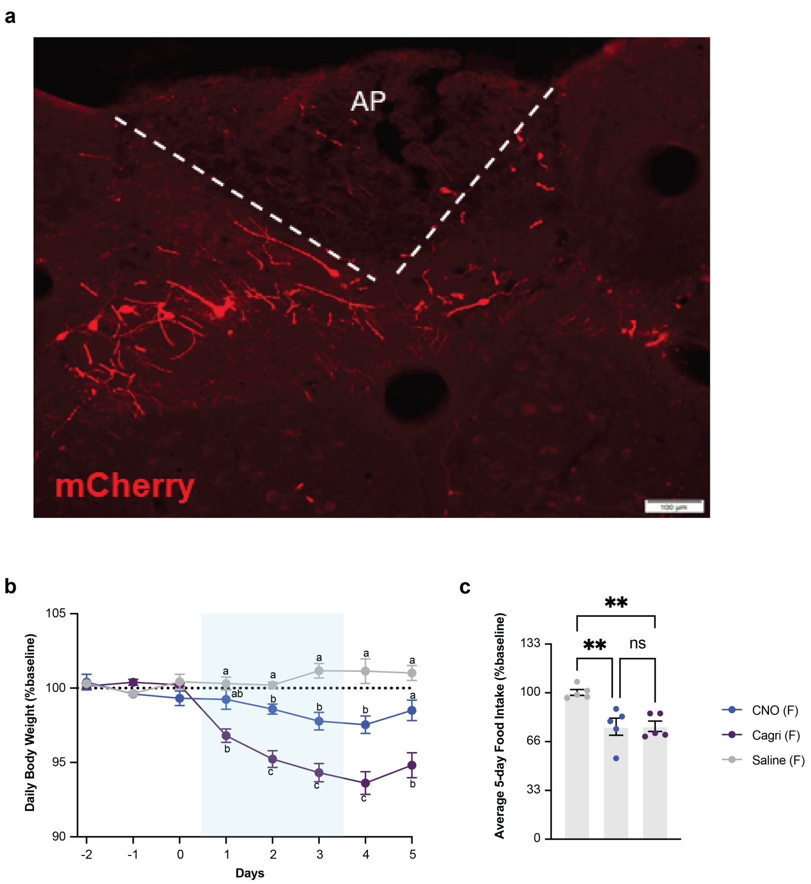

Supplementary Figure 16. Suppression of food intake and body weight by DREADD-mediated activation of NTS Calcr neurons in rats. **a**, representative image of mCherry-IR (red) in the DVC of a CalcrCre rat transduced in the NTS with AAV-DIO-hM3Dq-mCherry. AP is labelled and AP/NTS border is indicated by dashed while lines. Scale bar=200 um. **b**, Body weight (normalized to baseline) for CalcrNTS-Dq rats (n=5) treated with saline, cagrilintide (10 nmol/kg, sc, qd), or CNO (1 mg/kg, IP bid). **c**, Average food intake over 5 days (normalized to baseline daily intake over the study period).

Legends for Supplementary Tables

Supplementary Table 1. Number of animals per treatment for each species.

Supplementary Table 2. Number of neuronal and glia cells per treatment for each species.

Supplementary Table 3. Tabulated overview of all single-nucleus sequencing meta data for each species.

Supplementary Table 4. List of 100 genes targeted in the spatial transcriptomics imaging of the rat DVC. Each item includes the catalogue number of the manufacturer for the probe, the species, the ENSEMBL ID and the canonical gene name. Two items (*Lepr* and *Foxj1*) are custom probes.

Supplementary Table 5. Spatial enrichment of each neuronal cell type to regions in the DVC (AP, NTS, or DMV) denominated by the -log10 p-value for the respective cell type to be enriched in the region. Note: Sero0.0 has been excluded from the report because it was found to map outside of the anatomical area that was covered by spatial transcriptomics and is therefore false positive in this analysis.

Supplementary Table 6. Genetic enrichment (ESμ) values for each cell type and species for Calcr. Values above the threshold (>0.8) are highlighted.

Supplementary Table 7. Tabulated results of quantified IHC sections of the mouse DVC. Results are split for the AP and NTS and contain the absolute number and fraction (in %) of counted cells for each animal. Mice were labelled with Glp1r-Cre crossed to a tdTomato Cre-reporter line and stained for Calcr expression to quantify co-expression.

Supplementary Table 8. Tabulated results of quantified IHC sections of the mouse DVC. Results are split for the AP and NTS and contain the absolute number and fraction (in %) of counted cells for each animal. Mice were labelled with Calcr-Cre crossed to a tdTomato Cre-reporter line and stained for Glp1r expression to quantify co-expression.

Supplementary Table 9. Differential gene expression analysis results of rat bulk RNA-seq across all treatments. *P*-values were adjusted for multiple-testing using Benjamini-Hochberg FDR correction.

Supplementary Table 10. Gene ontology (GO) enrichment analysis of differentially expressed genes in rat bulk RNA-seq across all treatments. Enrichment was carried out on only the significantly regulated genes (*P*-adj < 0.05) using all tested genes as background. GO terms with a Benjamini-Hochberg adjusted *P*-values < 0.05 are reported

Supplementary Table 11. Cell population specific differential gene expression analysis results of rat and mice across all available treatment. *P*-values were adjusted separately within each species-treatment-

cell type contrast using Benjamini-Hochberg FDR correction. Genes with an adjusted p-value < 0.05 were concatenated and shown

Supplementary Table 12. Cell population specific differential gene expression analysis results of DIO mice vs. chow mice. *P*-values were adjusted separately for each cell type using Benjamini-Hochberg FDR correction. Genes with an adjusted *P*-value < 0.05 were concatenated and shown.
