# Supplementary material for: A cross-species atlas of the dorsal vagal complex reveals neural mediators of the effects of cagrilintide on energy balance
Source: Nat Metab. 2026 Jun 8;8(6):1350–67. doi: 10.1038/s42255-026-01539-3 (PMC13303089; doi:10.1038/s42255-026-01539-3)
Supplement: Supplementary file 2 — Reporting Summary [file 42255_2026_1539_MOESM2_ESM.pdf]

nature portfolio

Corresponding author(s): Tune H Pers

Last updated by author(s): Dec 13, 2024

Reporting Summary

Nature Portfolio wishes to improve the reproducibility of the work that we publish. This form provides structure for consistency and transparency in reporting. For further information on Nature Portfolio policies, see our Editorial Policies and the Editorial Policy Checklist.

Statistics

For all statistical analyses, confirm that the following items are present in the figure legend, table legend, main text, or Methods section.

n/a	Confirmed	
☐	☒	The exact sample size (*n*) for each experimental group/condition, given as a discrete number and unit of measurement
☐	☒	A statement on whether measurements were taken from distinct samples or whether the same sample was measured repeatedly
☐	☒	The statistical test(s) used AND whether they are one- or two-sided<br>*Only common tests should be described solely by name; describe more complex techniques in the Methods section.*
☐	☒	A description of all covariates tested
☐	☒	A description of any assumptions or corrections, such as tests of normality and adjustment for multiple comparisons
☐	☒	A full description of the statistical parameters including central tendency (e.g. means) or other basic estimates (e.g. regression coefficient) AND variation (e.g. standard deviation) or associated estimates of uncertainty (e.g. confidence intervals)
☐	☒	For null hypothesis testing, the test statistic (e.g. *F*, *t*, *r*) with confidence intervals, effect sizes, degrees of freedom and *P* value noted<br>*Give P values as exact values whenever suitable.*
☒	☐	For Bayesian analysis, information on the choice of priors and Markov chain Monte Carlo settings
☒	☐	For hierarchical and complex designs, identification of the appropriate level for tests and full reporting of outcomes
☐	☒	Estimates of effect sizes (e.g. Cohen's *d*, Pearson's *r*), indicating how they were calculated

Our web collection on statistics for biologists contains articles on many of the points above.

Software and code

Policy information about availability of computer code

Data collection	https://github.com/perslab/ludwig-coester-gordian-2025
Data analysis	https://github.com/perslab/ludwig-coester-gordian-2025

For manuscripts utilizing custom algorithms or software that are central to the research but not yet described in published literature, software must be made available to editors and reviewers. We strongly encourage code deposition in a community repository (e.g. GitHub). See the Nature Portfolio guidelines for submitting code & software for further information.

Data

Policy information about availability of data

All manuscripts must include a data availability statement. This statement should provide the following information, where applicable:

- Accession codes, unique identifiers, or web links for publicly available datasets
- A description of any restrictions on data availability
- For clinical datasets or third party data, please ensure that the statement adheres to our policy

Single-nucleus RNA-seq (E-MTAB-16929) and bulk RNA-seq (E-MTAB-16870) data are available in EMBL-EBI BioStudies. Spatial transcriptomics data are available at https://zenodo.org/records/19368177. All code used to analyse the data is available at https://github.com/perslab/ludwig-coester-gordian-2025.

Research involving human participants, their data, or biological material

Policy information about studies with human participants or human data. See also policy information about sex, gender (identity/presentation), and sexual orientation and race, ethnicity and racism.

Reporting on sex and gender	The study includes postmortem human brain samples (caudal medulla) obtained from the Edinburgh Brain Bank. These samples include both males and females, but specific sex-disaggregated analyses were not conducted due to the small sample size (n=5). The study focuses on molecular and cellular characteristics rather than gender-based comparisons.
Reporting on race, ethnicity, or other socially relevant groupings	Race, ethnicity, or other socially relevant groupings were not considered or reported in this study. The focus of the study was solely on postmortem brain tissue, and these variables were not relevant to the research question.
Population characteristics	The human brain samples consist of postmortem tissue blocks covering the caudal medulla, obtained from the Edinburgh Brain Bank. The sample includes both males and females (n=5). Genotypic or demographic information was not collected, as the study's primary goal was to characterize molecular features in the brain.
Recruitment	The human brain tissue samples were not actively recruited but were obtained postmortem through collaboration with the Edinburgh Brain Bank. The samples were provided following all ethical and legal guidelines in the UK and Denmark. No recruitment bias or self-selection applies to this study, as it solely used archived samples.
Ethics oversight	The human brain samples were obtained in collaboration with Professor Colin Smith from the Edinburgh Brain Bank. All procedures adhered to the legal and ethical guidelines in the UK and Denmark.

Note that full information on the approval of the study protocol must also be provided in the manuscript.

Field-specific reporting

Please select the one below that is the best fit for your research. If you are not sure, read the appropriate sections before making your selection.

☒ Life sciences ☐ Behavioural & social sciences ☐ Ecological, evolutionary & environmental sciences

For a reference copy of the document with all sections, see nature.com/documents/nr-reporting-summary-flat.pdf

Life sciences study design

All studies must disclose on these points even when the disclosure is negative.

Sample size	Sample sizes for single-cell transcriptomics experiments were determined based on prior experiences aimed at identifying cell populations exhibiting changes in gene expression levels and transcriptionally inferred neuronal activity (https://www.nature.com/articles/s42255-021-00363-1). Sample sizes for DREADD experiments were determined based on previous work by Prof. Martin G. Myers, Jr. (co-author) and were informed by studies such as https://pmc.ncbi.nlm.nih.gov/articles/PMC8405610/ and https://www.nature.com/articles/s42255-021-00363-1.
Data exclusions	Generally, no data points were excluded except in the following cases:<br><br>Injection placement: Animals with inadequate transduction of the AP or misplaced injections (assessed via dsRed staining for CalcrCre-hM3Dq brains and GFP fluorescence for shPrlh brains) were excluded.<br>Single-cell data: In differential expression analysis, animal and cell population combinations with fewer than five cells were excluded from the pseudobulk gene expression matrix constructed using DESeq2 v1.30.1.<br><br>snPrlh knock-down: Only samples with successful bilateral injections were kept.
Replication	Replication was achieved by:<br><br>Validating the most important cell population-specific markers using immunohistochemistry.<br>Confirming key transcriptional findings for the prlh gene using independent cohorts with bulk RNA-sequencing and single-cell sequencing approaches.
Randomization	Mice: Prior to study initiation, diet-induced obese mice were randomized into groups based on body weight. For the acute study, mice received a single subcutaneous injection of cagrilintide (10 nmol kg^{-1}), AM1213 (30 nmol kg^{-1}), or vehicle, administered 0–1 h after light onset. For the subchronic study, mice were dosed daily with the same treatments for seven days. A weight-matched group received restricted food to match the cagrilintide group's weight loss. Animals were euthanized 3–4 h after the final dose for brain dissection.<br>Rats: Diet-induced obese rats were similarly randomized by body weight. For the acute study, rats received a single injection of cagrilintide (3 nmol kg^{-1}) or vehicle, administered 0–1 h after light onset. For the subchronic study, rats received daily doses of cagrilintide or vehicle for seven days. A weight-matched group was included via daily food restriction. Brains were excised after the final dose and dissected for analysis.
Blinding	Blinding is not typically used in mouse experiments involving single-cell sequencing, DREADDs (Designer Receptors Exclusively Activated by Designer Drugs), or gene knockdown for the following reasons:<br><br>Objective and High-Throughput Nature of Data Analysis:

Downstream analyses in these experiments, such as single-cell RNA or ATAC sequencing, are data-driven and performed using computational pipelines. These pipelines are objective and do not require subjective interpretation. Thus, the need for blinding is minimized because bias is unlikely to be introduced at the analysis stage.

Clear and Distinct Experimental Groups:
In experiments involving DREADDs or gene knockdown, experimental groups are clearly defined by genetic modifications or viral transductions. Misclassification or bias is less of a concern compared to more subjective behavioral or physiological assays where blinding is critical to avoid human bias during data collection or interpretation.

Reporting for specific materials, systems and methods

We require information from authors about some types of materials, experimental systems and methods used in many studies. Here, indicate whether each material, system or method listed is relevant to your study. If you are not sure if a list item applies to your research, read the appropriate section before selecting a response.

Materials & experimental systems

n/a	Involved in the study
☐	☒ Antibodies
☒	☐ Eukaryotic cell lines
☒	☐ Palaeontology and archaeology
☐	☒ Animals and other organisms
☒	☐ Clinical data
☒	☐ Dual use research of concern
☒	☐ Plants

Methods

n/a	Involved in the study
☒	☐ ChIP-seq
☒	☐ Flow cytometry
☒	☐ MRI-based neuroimaging

Antibodies

Antibodies used

DsRed (Rabbit) Takara 632496; RRID:AB_10013483
FOS Immunofluorescence (Rabbit) Cell Signaling 2250; RRID:AB_2247211

Validation

DsRed, https://www.takarabio.com/products/antibodies-and-elisa/fluorescent-protein-antibodies/red-fluorescent-protein-antibodies?srsltid=AfmBOopvfOnknqe9bAm1pvlpnPzyifCausM9fQhYGqDomijOmxyvM-6i
https://www.cellsignal.com/products/primary-antibodies/c-fos-9f6-rabbit-mab/2250?srsltid=AfmBOopCu8plHpxe4gHXnVNGONNiI79a_8hMQZSijlynae-MvR8PrdQC

Animals and other research organisms

Policy information about studies involving animals; ARRIVE guidelines recommended for reporting animal research, and Sex and Gender in Research

Laboratory animals

1. CBMR: For spatial transcriptomics, 8-week-old male Sprague Dawley rats were used. No ethical permission was required from the Danish Animal Experiments Inspectorate because the animals were euthanized without undergoing experimental procedures.
2. Novo Nordisk A/S: Male Sprague Dawley rats aged 25 weeks were used for cagrilintide treatment and bulk RNA-sequencing. Ethical permission was obtained from the Danish Animal Experiments Inspectorate.
3. University of Michigan: Male CalcR-Cre rats aged 30 weeks were used for DREADD-mediated activation of AP Calcr-neurons. Ethical approval was granted by the University of Michigan Animal Care and Use Committee (USA), adhering to the guidelines of the Association for the Assessment and Accreditation of Laboratory Animal Care and NIH standards.
4. University of Michigan: Male Sprague Dawley rats aged 27 weeks underwent stereotaxic surgeries (shPrlh). Ethical approval was granted by the University of Michigan Animal Care and Use Committee (USA), adhering to the guidelines of the Association for the Assessment and Accreditation of Laboratory Animal Care and NIH standards.
5. Novo Nordisk A/S: Male Sprague Dawley rats aged 26 weeks were used in vivo for cagrilintide treatment studies. Ethical permission was obtained from the Danish Animal Experiments Inspectorate.
Novo Nordisk A/S: Male C57BL/6J mice aged 27 weeks were used for in vivo cagrilintide treatment studies. Ethical permission was obtained from the Danish Animal Experiments Inspectorate.

Wild animals

Not applicable as no wild animals were used in the studies described.

Reporting on sex

The sex of the animals used in all experiments is specified. Where applicable, both male and female animals were included (e.g., rhesus macaque studies), ensuring sex was considered in the study design.

Field-collected samples

Not applicable as no samples were collected from the field.

Ethics oversight

1. CBMR: Ethical permission was not required as the animals were euthanized without experimental procedures.
2. Novo Nordisk A/S and University of Michigan: Ethical approvals were obtained from respective national and institutional committees, with adherence to applicable regulations and guidelines.
3. Oregon Health and Science University: Procedures with rhesus macaques were conducted in accordance with the Institutional Animal Care and Use Committee (IACUC) of the ONPRC. Compliance with the Animal Welfare Act and USDA regulations was ensured.

Note that full information on the approval of the study protocol must also be provided in the manuscript.

Plants

Seed stocks	Not applicable. No seed stocks or plant material were used in this work.
Novel plant genotypes	Not applicable. No novel plant genotypes were produced or analyzed in this work.
Authentication	Not applicable. No plant genotypes or seed stocks were utilized, and therefore, no authentication procedures were required.
